# Histone modification dynamics as revealed by multicolor immunofluorescence-based single-cell analysis

**DOI:** 10.1242/jcs.243444

**Published:** 2020-07-21

**Authors:** Yoko Hayashi-Takanaka, Yuto Kina, Fumiaki Nakamura, Leontine E. Becking, Yoichi Nakao, Takahiro Nagase, Naohito Nozaki, Hiroshi Kimura

**Affiliations:** 1 Graduate School of Bioscience and Biotechnology, Tokyo Institute of Technology, 4259 Nagatsuta, Midori-ku, Yokohama 226-8501, Japan; 2 Graduate School of Frontier Biosciences, Osaka University, 1-3, Yamadaoka, Suita 565-0871, Japan; 3 Graduate School of Advanced Science and Engineering, Waseda University, 3-4-1 Okubo, Shinjuku-ku, Tokyo 169-8555, Japan; 4 Marine Animal Ecology Group, Wageningen University & Research, PO Box 338, Bode 36, 6700 AH Wageningen, The Netherlands; 5 Kazusa DNA Research Institute, Chiba 292-0818, Japan; 6 Mab Institute Inc, 2070-11, Osegi, Iida 395-0157, Japan; 7 Cell Biology Center, Institute of Innovative Research, Tokyo Institute of Technology, 4259 Nagatsuta, Midori-ku, Yokohama 226-8503, Japan

**Keywords:** Chemical biology, Chromatin, Epigenetics, Histone modification, Monoclonal antibody

## Abstract

Post-translational modifications on histones can be stable epigenetic marks or transient signals that can occur in response to internal and external stimuli. Levels of histone modifications fluctuate during the cell cycle and vary among different cell types. Here, we describe a simple system to monitor the levels of multiple histone modifications in single cells by multicolor immunofluorescence using directly labeled modification-specific antibodies. We analyzed histone H3 and H4 modifications during the cell cycle. Levels of active marks, such as acetylation and H3K4 methylation, were increased during the S phase, in association with chromatin duplication. By contrast, levels of some repressive modifications gradually increased during G2 and the next G1 phases. We applied this method to validate the target modifications of various histone demethylases in cells using a transient overexpression system. In extracts of marine organisms, we also screened chemical compounds that affect histone modifications and identified psammaplin A, which was previously reported to inhibit histone deacetylases. Thus, the method presented here is a powerful and convenient tool for analyzing the changes in histone modifications.

## Abstract (precis)

**Highlighted Article:** Profiling histone modification dynamics during the cell cycle, in response to lysine demethylase overexpression and upon chemical inhibitor treatments.

## INTRODUCTION

In eukaryotes, DNA is wrapped around eight histone proteins to form nucleosomes, which are the basic units of chromatin. Post-translational modifications of histones play a crucial role in gene regulation by altering chromatin structure and/or recruiting reader proteins (Jenuwein and Allis, 2001; Kouzarides, 2007). Site-specific acetylation and methylation of histone H3 lysine (H3K) residues are associated with gene activation and silencing (Lawrence et al., 2016) and their genome-wide distributions have been analyzed in various cell types (Kimura, 2013). Some modifications are stable epigenetic marks, whereas others turnover rapidly and/or exhibit dynamic changes in response to external and internal stimuli (McBrian et al., 2013; Niu et al., 2015) and during the cell cycle (Black et al., 2012). For example, H4 acetylation and H3 phosphorylation drastically increase during the S and M phases of the cell cycle, respectively (Chahal et al., 1980; Gurley et al., 1975). Recent analyses have also revealed that histone methylation states fluctuate during the cell cycle (Bar-Ziv et al., 2016; Petruk et al., 2013; Xu et al., 2012), possibly reflecting the balance between methylation and demethylation enzymes (Greer and Shi, 2012; Shmakova et al., 2014).

Histone modifications have traditionally been detected by radiolabeling and immunoassay using specific antibodies. Recently, mass spectrometry (MS) has emerged as a powerful method for comprehensively revealing multiple modifications in a single histone molecule, as well as their turnover rates (Zee et al., 2010a). These techniques require a homogenous population of a relatively large number of cells, often with cell cycle synchronization. By contrast, single-cell analysis based on flow cytometry and microscopy can be applied to a heterogeneous cell population, although the quality and accuracy of the results depend on the properties of the antibodies. A recent high-throughput microscopy-based single-cell assay has revealed differences in histone modifications between normal and cancer cells (Zane et al., 2017).

In this study, we report a simple multicolor immunofluorescence technique to reveal histone modifications in single cells using directly labeled modification-specific antibodies (Chandra et al., 2012; Hayashi-Takanaka et al., 2011, 2015; Kimura et al., 2008). With image analysis, we profiled levels of up to four histone modifications in single cells. We applied this method to the systematic analysis of the dynamic changes in histone modifications during the cell cycle and the targets of histone lysine demethylases (KDMs). Furthermore, we screened the extracts of marine organisms and identified a compound that inhibits histone deacetylase.

## RESULTS

### Multicolor immunofluorescence-based single-cell analysis

**Fig. 1.**
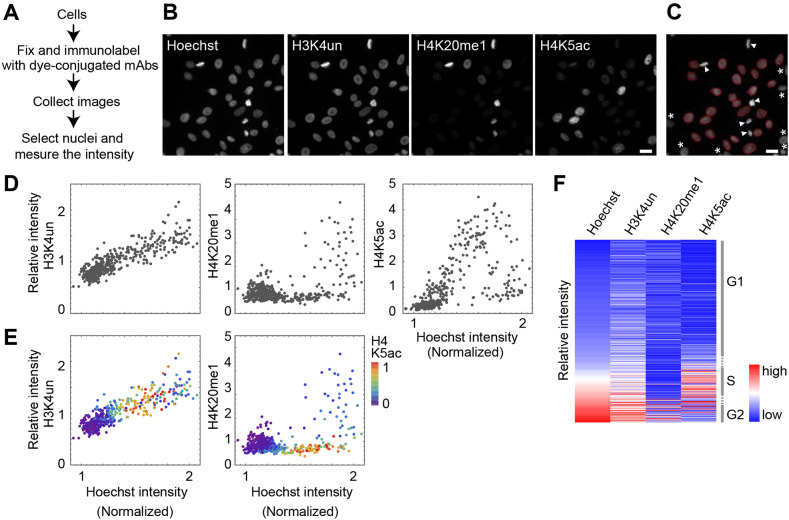
**Single-cell multicolor immunofluorescence analysis.** (A) Experimental flow of the analysis. (B-F) HeLa cells were stained with Hoechst 33342 and antibodies specific for H3K4un (Alexa Fluor 488), H4K20me1 (Cy3) and H4K5ac (Cy5). (B) Microscopy images in four channels. (C) The areas of individual nuclei were determined by thresholding using Hoechst signals. Cells with incomplete nuclear shape (asterisks) were excluded from the analysis. Mitotic chromosomes with smaller areas than interphase nuclei (arrowheads) were also excluded from the analysis. (D) Typical two-dimensional graphs plotting relative intensity of histones against Hoechst intensity. The average intensity was set to 1 on the *y*-axis. (E) Typical three-dimensional graphs. Levels of replication marker H4K5ac are shown with the rainbow color code. (F) Typical heat maps. The relative intensities in single cells are color-coded and sorted using Hoechst signal intensity (lowest at the top and highest at the bottom). The positions of the cell cycle phase are indicated on the right. Cell numbers for D-F: 450. Scale bars: 20 μm.

To analyze the global levels of multiple modifications in single cells, HeLa cells were grown on coverslips, fixed and immunolabeled with various antibodies directly conjugated to fluorescent dyes. Fig. 1 illustrates the scheme (Fig. 1A) and a typical example of the analysis (Fig. 1B-F) with antibodies directed against unmodified histone H3 at lysine 4 (H3K4un; Alexa Fluor 488), monomethylated histone H4 at K20 (H4K20me1; Cy3), and acetylated histone H4 at K5 (H4K5ac; Cy5). Cells were also stained with Hoechst 33342 to detect DNA. Fluorescence images were acquired using a wide-field fluorescence microscope with the standard filter sets (Fig. 1B), and the sum of intensities (average intensity number of pixels in the nuclear area) in each nucleus was measured (Fig. 1C). The partial nuclei at the edge of the field were removed because the whole nuclear area was used for determining the total signal intensity. To examine variations within a cell population, the relative signal intensity in each nucleus was obtained by normalization against the average of all nuclei.

Total signal intensities of various antibodies and the Hoechst dye shown in a dot plot (Fig. 1D) reveal the distribution of histone modification levels in different phases of the cell cycle, as judged by Hoechst total intensity. The level of H3K4un simply increased with cell cycle progression, which is consistent with the increase in the number of nucleosomes during DNA replication, as most of the H3K4 (∼90%) was unmodified (Huang et al., 2015). Empirically, analysis of approximately 400-500 nuclei was usually sufficient to obtain feasible and reproducible results (Fig. S1A). The level of H4K5ac modification was highest during the S phase, consistent with the diacetylation of histone H4 at K5 and K12 in a deposition complex (Allis et al., 1985; Chicoine et al., 1986; Sobel et al., 1995). The enrichment of H4K5ac in S phase cells was further confirmed by double staining using 5-ethynyl-2′-deoxyuridine (EdU), which was incorporated into the replicated DNA via pulse-labeling immediately before cell fixation (Fig. S1B,C). Because H4K5ac could be a good S-phase marker, indicating H4K5ac levels in the color scale facilitated the identification of S-phase cells (Fig. 1E). In contrast to H3K4un and H4K5ac, a drastic increase in H4K20me1 levels during G2 was observed (Fig. 1D,E). This was also consistent with previous reports demonstrating the fluctuation in H4K20me1 levels during the cell cycle, increasing at G2 and M phases (Pesavento et al., 2008; Rice et al., 2002; Sato et al., 2016). These data were consistent with profiles obtained using flow cytometry (Fig. S2A-C) and DNA staining with propidium iodide (PI), an intercalator without sequence specificity, instead of Hoechst binding to A/T-rich sequences (Fig. S2D,E). Therefore, the dot plots well represented the distribution of histone modifications during the cell cycle. However, we excluded mitotic cells from routine analysis for two reasons: First, mitotic chromosomes generally occupied smaller areas than occupied by nuclei at the nuclear focal plane, resulting in approximately 13% lower total intensities (Fig. S2F). Second, most modification-specific antibodies used in this study are sensitive to the phosphorylation of neighboring serine/threonine residues that occurs during mitosis (Kimura et al., 2008; Hayashi-Takanaka et al., 2011; Kimura, 2013), which complicates data interpretation. Moreover, four new antibodies specific for histones unmodified on K9 and K27 (H3K9un and H3K27un) or modified by monomethylation (H3K27me1) or dimethylation (H3K27me2) of K27, did not bind to the target modifications when the next amino acid residues (S10 and S28) were phosphorylated (Fig. S3). The H3K27me1-specific antibody was also sensitive to R26 methylation and citrullination; however, these R26 modifications are not abundant (Guertin et al., 2014) and antagonize K27 methylation (Clancy et al., 2017), so their contribution would be minor.

To compare more than two modifications with the Hoechst signal, a heat map was used (Fig. 1F) in which each row corresponded to a single cell. When aligned in order of Hoechst intensity from the lowest (from the top to the middle and bottom rows in Fig. 1F, corresponding to G1, S and G2, respectively), the higher signals of H4K5ac and H4K20me1 appeared in the middle and bottom rows, respectively, consistent with cell cycle progress. Thus, once the immunostaining and intensity measurements are performed, an appropriate visualization method can be chosen, depending on the purpose of analysis.

### Evaluating the robustness of the assay system

**Fig. 2.**
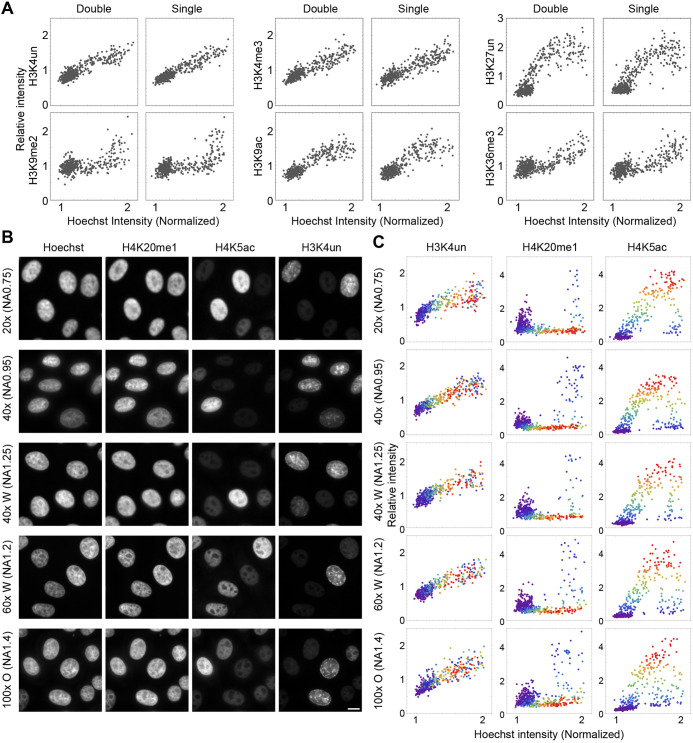
**Effects of antibody staining and imaging conditions using different objective lenses.** (A) Comparison of single and double antibody staining. Fixed and permeabilized HeLa cells were stained with a single antibody or a pair of antibodies, using a combination of H3K4un and H3K9me2, H3K4me3 and H3K9ac, or H3K27un and H3K36me3. The profiles of histones and Hoechst intensities are shown. Cell numbers: 420. (B,C) Effects of the numerical aperture and magnification of the objective lens on the intensity profiles. Fixed and permeabilized HeLa cells were stained using Hoechst 33342 and antibodies specific for H3K4un (Alexa Fluor 488), H4K20me1 (Cy3) and H4K5ac (Cy5). Images were collected using five different lenses: 20× (dry, NA 0.75), 40× (dry, NA 0.95), 40× (water-immersion, NA 1.25), 60× (water-immersion, NA 1.2), and 100× (oil-immersion, NA 1.4). Typical images are shown in B. Signal intensities of histones plotted against Hoechst intensity and H4K5ac levels are shown in C, with the rainbow color code as in Fig. 1E. Cell numbers: 450. Scale bar: 10 μm.

To evaluate the robustness of the assay, we examined the effects of antibody staining conditions using objective lenses with different numerical apertures (NAs). We first analyzed whether the antibody concentration is crucial for obtaining quantitative data. HeLa cells were fixed, permeabilized and incubated with serially diluted series of fluorescence-labeled antibodies (0.25-4 μg/ml). Immunofluorescence images were collected using the same exposure settings as those for quantitative analyses. Fluorescence intensities were lower in samples stained with lower antibody concentrations but the histone modification profiles were highly similar, regardless of the antibody concentration tested (Fig. S4). Next, we compared the profiles for single and double antibody staining to determine whether one antibody interferes with another via steric hindrance during multiple labeling (Fig. 2A). We chose combinations of two modifications that are relatively close and co-exist on a single histone molecule, such as H3K4un and H3K9me2 (Huang et al., 2015), H3K4me3 and H3K9ac (Kimura et al., 2008) and H3K27un and H3K36me3 (Yuan et al., 2011). For all tested combinations, the double labeling profiles were essentially the same as single labeling profiles (Fig. 2A), suggesting no or little effect of one antibody on the binding of another under the given conditions. This is probably because antibody binding was not at a saturated level (as seen in Fig. S4). Therefore, we conclude that the antibody concentration and combination are not crucial for the assay.

**Fig. 3.**
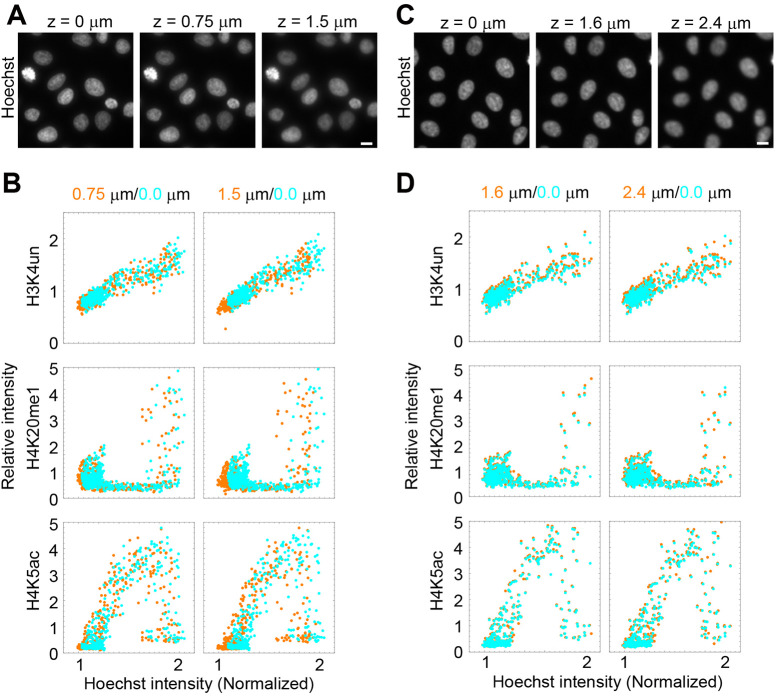
**Effects of focal shifts on intensity profiling.** Fixed and permeabilized HeLa cells were stained with Hoechst 33342 and antibodies specific for H3K4un (Alexa Fluor 488), H4K20me1 (Cy3) and H4K5ac (Cy5). (A,C) Fluorescence images were collected in a z-stack using an NA 0.95 40× objective lens with 0.25 μm intervals (A) and an NA 0.75 20× lens with 0.4 μm intervals (C). Hoechst images were taken at different planes in A and C. Distances from the focal plane are indicated. (B,D) Intensities in the different focal planes were measured, normalized and plotted. The intensities in the focal and another plane are indicated in cyan and orange, respectively. Cell numbers: 450. Scale bars: 10 μm.

Furthermore, we investigated the suitability of objective lenses with different NAs for this assay (Fig. 2B,C). In principle, compared with a low NA objective lens, a high NA lens has superior optical resolution and brightness but is less tolerant to focal shifts, which can be a concern when many nuclei with slightly different focal planes are analyzed. When different objective lenses with NAs of 0.75-1.4 were compared, the intensity profiles were similar for lenses with NAs 0.75-1.25 (20× to 60×), but the Hoechst intensities were distributed more broadly when a 100× NA 1.4 oil-immersion lens was used (Fig. 2B,C). These data suggest that a lens with the highest NA (1.4) may not be appropriate for this assay, even though the spatial distribution of individual modifications is better visualized. In contrast, the spatial resolution was poor with a 20× NA 0.75 lens (Fig. 2B). We also analyzed the effect of focal shifts on the quantified data using 40× NA 0.95 and 20× NA 0.75 objective lenses (Fig. 3). With the 40× NA 0.95 lens, a substantial difference in the intensity distribution was observed between the equatorial focal plane and a plane 1.5 μm above the focus (Fig. 3A,B). The difference was small in a plane 0.75 μm above the focus, suggesting that submicrometer focus shifts are tolerant for analysis using a 40× NA 0.95 lens. In contrast, using a 20× NA 0.75 lens, the intensity distributions of a plane 2.4 μm above the focus were almost the same as those of the focal plane, which is consistent with the deeper focal depth of a low NA lens (Fig. 3C,D). Therefore, a 20× (NA 0.75) lens appears to be suitable for a high-throughput and robust analysis; however, a primary drawback is its poor resolution. Therefore, we routinely used a 40× dry NA 0.95 lens because the intranuclear distribution of individual modifications can be reasonably well visualized, submicrometer focal shifts are allowed and it is convenient to use without an immersion medium.

### Histone modifications during the cell cycle

**Fig. 4.**
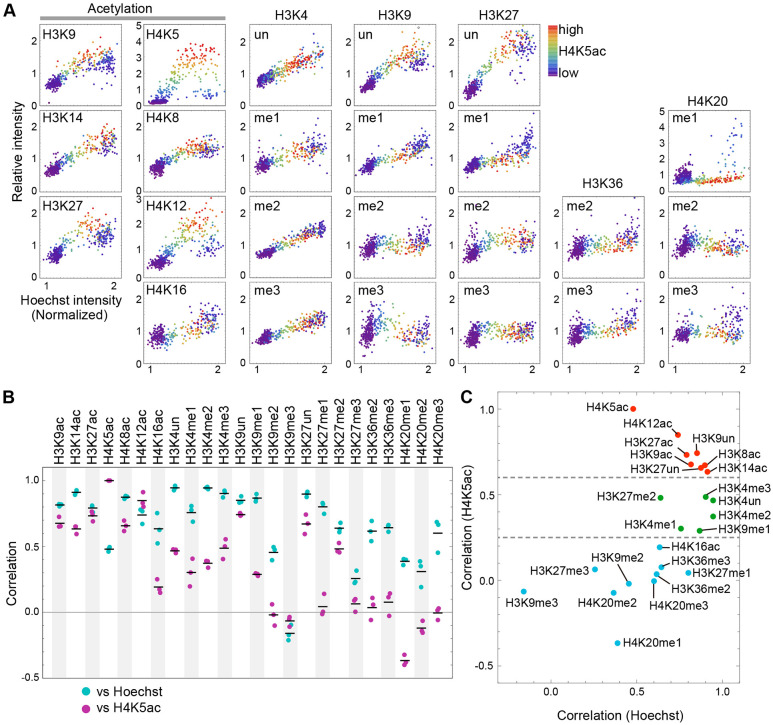
**Detection of histone modification levels during the cell cycle in hTERT-RPE1 cells.** (A) Acetylation and mono- (me1), di- (me2) and trimethylation (me3) of histones H3 and H4 during the cell cycle; un, unmodified. Cell numbers: 450. (B) Correlation between target modification and Hoechst or H4K5ac signals. The values from three biologically independent replications (dots) and their averages (bar) are plotted. (C) Correlation function for Hoechst intensity and histone modification, based on B, plotted in two dimensions.

We first applied the multicolor immunofluorescence analysis to reevaluate changes in various histone modifications during the cell cycle (Fig. 4A). hTERT-RPE1 cells were fixed and stained with an Alexa Fluor 488-labeled modification-specific antibody, together with Cy5-labeled H4K5ac-specific antibody and Hoechst 33342. In dot plots, cells in the S phase were highlighted by higher H4K5ac levels using the rainbow color scale. To quantitatively compare the histone modification profiles with the DNA content (Hoechst) and S-phase enrichment (H4K5ac), Pearson correlation coefficients were calculated using three biologically independent experiments (Fig. 4B).

As a proof of concept, we first examined the levels of H3K4un and H4K12ac. As mentioned above, we expected high correlations between H3K4un and DNA content and between H4K12ac and H4K5ac. Indeed, H3K4un showed a strong correlation with the Hoechst signal (correlation factor, 0.95; Fig. 4B). The distribution profile of H4K12ac was similar to that of H4K5ac (Fig. 4A), with a high correlation (correlation factor, 0.85; Fig. 4B), consistent with their coexistence in newly assembled chromatin (Allis et al., 1985; Chicoine et al., 1986; Sobel et al., 1995). These data indicate that the analytical method presented here can be used to evaluate histone modification profiles during the cell cycle.

To overview the characteristics of individual modifications (Fig. 4A,B), we plotted their correlation with Hoechst 33342 and H4K5ac signals (Fig. 4C). Based on the correlations with H4K5ac, we classified histone modifications into three groups. The first group, showing high correlations with H4K5ac (>0.6), contained unmodified H3K9 and H3K27 (H3K9un and H3K27un) and all histone acetylations, except H4K16ac. These modifications also showed high correlations with Hoechst signal (>0.7). In single-cell dot plots (Fig. 4A), their signal intensities were clearly increased in S-phase cells. These data suggest that newly assembled histones are acetylated soon after DNA replication. The higher levels of these modifications during S phase than during G2 phase could be correlated with chromatin decondensation during or after DNA replication, as reported previously (Li et al., 1998). Levels of H3K9un and H3K27un also increased during the S phase, thus reflecting assembly of the unmodified form of H3 (Jasencakova et al., 2010). In contrast to H3K4un, the levels of H3K9un and H3K27un decreased during G2 phase, being reciprocal to the increase in the abundant methylation of lysine residues (e.g. H3K9me2 and H3K27me1, both occupying ∼36% of the H3) (Huang et al., 2015) from late S to G2 phase.

The second group, showing low to modest correlations with H4K5ac (0.25-0.6), contained all forms of H3K4, together with H3K9me1 and H3K27me2 (Fig. 4C). These H3K4 modifications also showed high correlations with Hoechst signal (>0.75), particularly H3K4me2 (0.94) and H3K4me3 (0.90). These data suggest that methylations of H3K4, which are associated with transcriptionally active chromatin (Bernstein et al., 2005; Kim et al., 2005; Roh et al., 2005), occur soon after DNA replication during the early S phase, consistent with the relatively rapid turnover rate of these methylations (Reverón-Gómez et al., 2018).

Because H3K9me1 is also enriched in the gene body of transcribed genes, chromatin containing this modification is likely to be replicated during early S phase, and it can be restored during S phase. This methylation is mediated through the G9a/GLP complex, which is also a methyltransferase responsible for H3K9me2 that is restored after the S phase (see below) (Alabert et al., 2015; Lin et al., 2016). H3K27me2 is an abundant modification, occupying ∼34% of H3 (Huang et al., 2015), and the polycomb repressive complex 2 acts as a methyltransferase for this modification as well as for H3K27me3 (Harutyunyan et al., 2019). The H3K27me2-containing chromatin might replicate earlier than H3K27me3 for earlier restoration.

The third group, showing little or anti-correlation with H4K5ac (<0.2), contained repressive marks and H3K36 methylation. In this group, correlations of the Hoechst signal with the constitutive (H3K9me3) and facultative (H3K27me3) heterochromatin marks were also low. Levels of modification decreased during the middle-to-late S phase and increased during G2 phase and the next G1 phase (Fig. 4A). In addition, levels of these modifications showed a broad distribution in G1 cells. This suggests that these modifications were not restored during the S and G2 phases within the same cell cycle and continued increasing during the next G1 phase. These observations are consistent with reports showing a transient decrease in the levels of these modifications during S phase, and their delayed restoration in the next cell cycle (Alabert et al., 2015; Reverón-Gómez et al., 2018; Xu et al., 2012; Zee et al., 2012). The level of H3K9me2 did not change much during most of the S phase and increased during late S and G2 phases (Fig. 4A), showing a modest correlation with the Hoechst signal (0.45), unlike H3K9me1. As mentioned above, the different restoration timing might reflect the different replication timing of H3K9me1- and H3K9me2-rich chromatin and/or a stepwise progression in the order of mono- and dimethylation mediated by the G9a/GLP complex. The restoration of H3K9me2 might continue throughout the next G1 phase (Fukuda et al., 2019).

Additional methylation marks, including H3K36me2, H3K36me3 and H3K27me1, behaved similarly to H3K9me2, but with slightly higher correlations with the Hoechst signal (>0.6). The delay in replication could partly explain the profile, although the mechanism remains unknown. H3K36me2 and H3K36me3 localized broadly to euchromatin and the gene body, respectively (Li et al., 2019; Zee et al., 2010b), and the replication timing of chromatin that harbors these modifications might not be as early as that of H3K4-associated chromatin. Because H3K27 is reportedly methylated (H3K27me1) in combination with H3K36 (Zheng et al., 2016), its dynamics could be similar to H3K36 methylation.

Unlike other acetylation marks, H4K16ac belonged to the third group, showing a lower correlation with H4K5ac (0.20). The level of H4K16ac increased throughout the S and G2 phases. Because H4K16ac is an abundant acetylation mark (∼30-40% of total H4 in HeLa cells) (Smith et al., 2003), its full restoration might require more time than other acetylation marks. The replication-uncoupled acetylation mechanism might also help regulate this aging-related acetylation (Dang et al., 2009). The abundance of H4K20me2 and H4K20me3 was gradually restored during late S phase to the next G1 phase, which is consistent with previous data (Pesavento et al., 2008; Yamamoto et al., 2015). Overall, the cell cycle analysis data based on multicolor immunofluorescence are in good agreement with previous findings, thus confirming the validity of this method.

### Targets of lysine demethylases

**Fig. 5.**
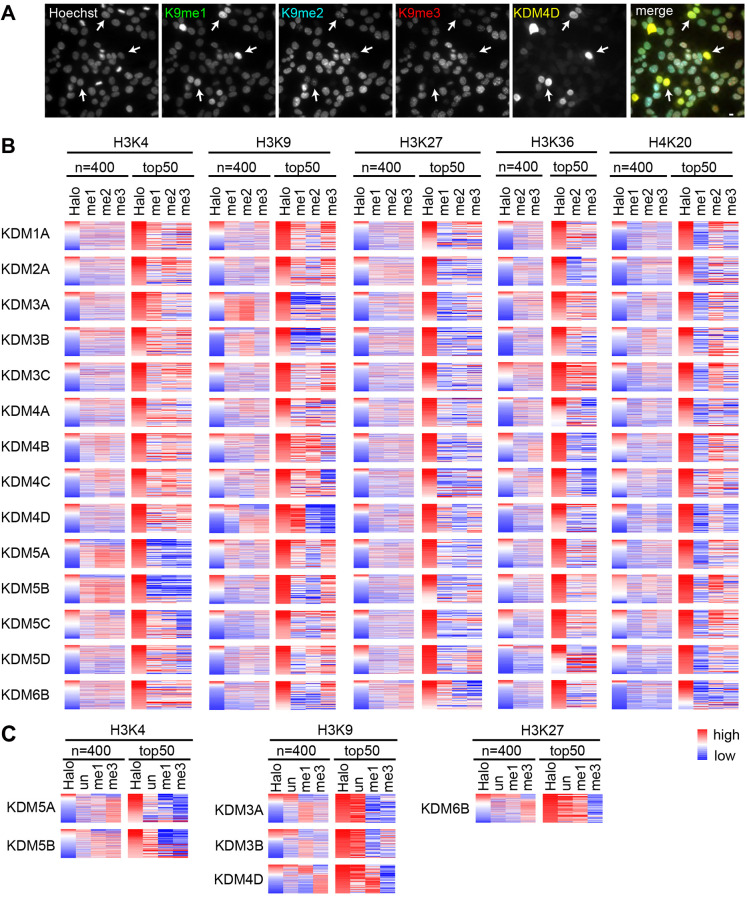
**Effect of histone demethylase on histone modifications in the 293T cells.** (A) Images of KDM-overexpressing cells. 293T cells were transiently transfected with various HaloTag-KDM expression vectors. After fixation, the cells were stained with Hoechst 33342, anti-HaloTag antibody and three anti-histone modification-specific antibodies. The example images are of cells that overexpressed KDM4D and were stained with anti-H3K9me1, H3K9me2, H3K9me3 and HaloTag antibodies. (B) Heat maps showing the three methylation levels (mono-, di-, and tri-) associated with HaloTag-KDM overexpression and sorted by HaloTag expression. The results of both whole cells (400 cells) and the top 50 cells expressing higher levels of HaloTag are shown. The decreased levels of methylation are shown in blue. (C) Effects of HaloTag-KDM expression on the levels of unmodified, mono- and trimethylated histone lysine residues. Scale bar: 10 μm.

**Fig. 6.**
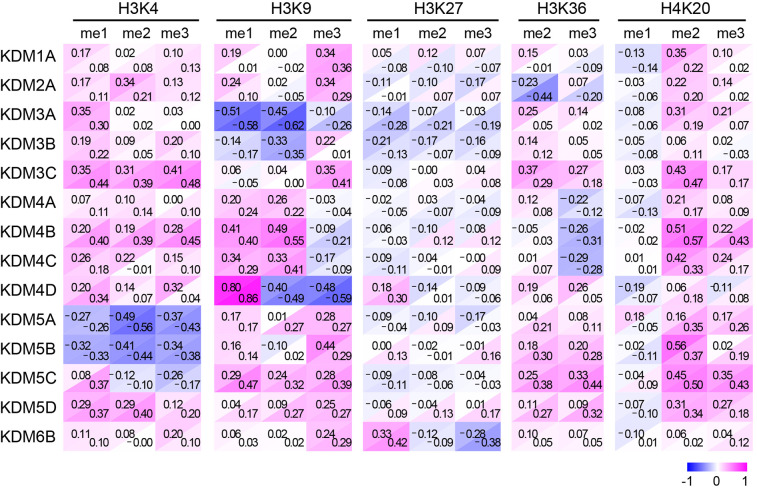
**Correlation between the intensities of HaloTag and histone modifications.** Data with red-blue background color were calculated from two independent experiments, based on Fig. 5B.

We applied the assay to analyze systematically the cellular targets of lysine demethylases (KDMs) by transiently expressing HaloTag-fusion proteins in 293T cells (Figs 5, 6). Cells grown on coverslips were transfected and then fixed the next day for staining with fluorescently (Alexa Fluor 488, Cy3, or Cy5) labeled antibodies directed against mono-, di- and trimethylated forms on the same lysine residue. The HaloTag-KDM was detected by HaloTag-specific rabbit polyclonal antibody with Alexa Fluor 750-labeled anti-rabbit IgG. Fig. 5A illustrates a typical example of cells that were transfected with a HaloTag-KDM4D expression vector and then stained with antibodies specific for H3K9me1 (Alexa Fluor 488), H3K9me2 (Cy3), H3K9me3 (Cy5) and HaloTag (Alexa Fluor 750), together with Hoechst 33342. The HaloTag-positive cells showed more intense signals for H3K9me1 and less intense signals for H3K9me2 and H3K9me3 (Fig. 5A, arrows), indicating that HaloTag-KDM4D removed methyl groups from H3K9me2 and H3K9me3, converting them to H3K9me1.

After quantifying the fluorescence signals of individual nuclei, the relative intensities were expressed as a heat map, which was aligned in order of the HaloTag level for 400 randomly selected cells (Fig. 5B; left columns for each methylation site). Because many cells did not express HaloTag-KDM or showed a low level of expression, the top 50 cells showing higher expression levels were zoomed-in (Fig. 5B; right columns). The decreased levels of methylation are shown in blue. To evaluate quantitatively the effect of KDM expression on individual histone modifications, Pearson correlations were calculated; the results of two independent experiments are shown in Fig. 6.

H3K4 was demethylated by KDM5 family members (Greer and Shi, 2012). Expression of KDM5A and KDM5B reduced the levels of all three methylation patterns (mono-, di- and tri-), as reported previously (Christensen et al., 2007), and KDM5C showed weak activity. We then stained transfected cells with H3K4un together with H3K4me1 and H3K4me3 in an attempt to see whether the increased level of H3K4un could be reciprocally observed in cells that lost methylation at H3K4 (Fig. 5C). In cells expressing HaloTag-KDM5A and HaloTag-KDM5B, the levels of H3K4un were moderately high but not the highest, possibly because methylated H3K4 occupies only ∼13% of the H3 (Huang et al., 2015); therefore, only a small increase in the level of H3K4un would be expected even if all of the methylated fraction changed to the unmodified state.

H3K9 was demethylated by various KDM families, including KDM3 and KDM4. The KDM3A and KDM3B enzymes preferentially reduced the levels of H3K9me1 and H3K9me2 more than that of H3K9me3 (Fig. 5B, Fig. 6). An increase in the level of H3K9un was reciprocally observed (Fig. 5C), consistent with the abundance of methylation (∼20% and ∼35% for mono- and dimethylation, respectively) similarly to and higher than that without methylation (∼21%) (Huang et al., 2015). Among the KDM4 family members, KDM4D demethylated both H3K9me2 and H3K9me3 to yield H3K9un and H3K9me1 (Fig. 5B,C).

H3K27me3 was demethylated by KDM6B overexpression (Fig. 5B, Fig. 6), consistent with previous reports (Agger et al., 2007; Chandra et al., 2012; Hong et al., 2007; Lan et al., 2007; Xiang et al., 2007). Levels of H3K27un and H3K27me1 were reciprocally increased in cells expressing KDM6B (Fig. 5B,C). H3K36 was demethylated by KDM2A and KDM4 family members. KDM2A showed preference for H3K36me2 over H3K36me3, whereas KDM4 showed the opposite trend (Hillringhaus et al., 2011; Tsukada et al., 2006). Methylation levels of H4K20 did not show a significant decrease with the KDMs used in this study. Overall, these results confirmed previous observations, further supporting the reliability of the assay system.

### Screening of marine organism extracts

**Fig. 7.**
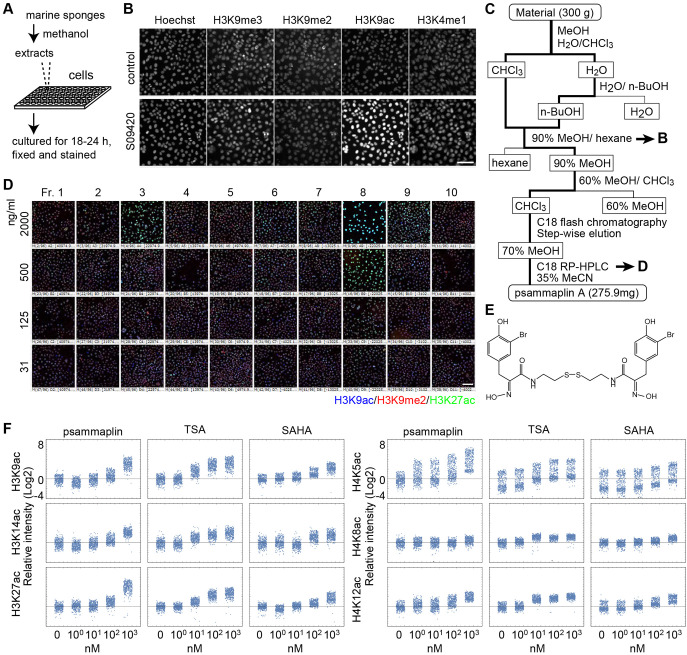
**Determination of HDAC activity in marine organism extracts.** (A) Scheme used for the screening of substances affecting histone modifications. (B) Initial screening using a cell-based assay. The fraction of the sponge *Aplysilla* sp. (S09420) increased the levels of H3K9ac marks. (C) Scheme used for the purification of psammaplin A from the marine sponge. (D) Assays using the dilution series of each fraction. (E) The structural formula of psammaplin A. (F) Effect of HDAC inhibitors, psammaplin A, TSA and SAHA on the levels of histone acetylation. The levels of H3K9, H3K14, H3K27, H4K5, H4K8 and H4K12 were investigated using tenfold serial dilutions. Cell numbers: 400. Scale bars: 100 μm.

We then applied the method for screening bioactive compounds from marine organisms (Hayashi-Takanaka et al., 2019). MDA-MB-231 breast cancer cells were grown in 96-well glass-bottom plates and cultured for 18-24 h with organic compounds extracted from marine organisms (Fig. 7A). The cells were then fixed and incubated with Hoechst 33342 and various histone modification-specific antibodies (Fig. S5). Among 3750 extracts tested, the hydrophobic extract (S09420) prepared from a marine sponge *Aplysilla* sp. collected at Chuuk, Federated States of Micronesia, markedly increased the levels of H3K9ac (Fig. 7B) and other acetylations (Fig. S5). To purify the compound responsible for the increase in H3K9ac level, the extract was fractionated by solvent partitioning and column chromatography (Fig. 7C). Levels of H3K9ac and H3K27ac were increased in cells treated with fraction 3 and fraction 8 through a C_18_ HPLC column (Fig. 7D). MS and NMR spectrometry (Fig. 7E; Fig. S6) revealed that both fractions contained psammaplin A, which was isolated from marine sponges including *Psammaplysilla* sp. (Arabshahi and Schmitz, 1987; Quiñoà and Crews, 1987; Rodriguez et al., 1987). Psammaplin A has been reported to inhibit the activity of DNA topoisomerase (Jiang et al., 2004), gyrase (Tabudravu et al., 2002), histone deacetylase (HDAC) and DNA methyltransferase *in vitro* (Piña et al., 2003).

We next compared the effects of psammaplin A on H3 and H4 acetylation with the commonly used HDAC inhibitors, trichostatin A (TSA) and suberoylanilide hydroxamic acid (SAHA) (Fig. 7F). Cells were incubated in serial dilutions of these compounds (0, 1, 10, 100 and 1000 nM) for 4 h and then fixed and stained with acetylation-specific antibodies. After measuring the fluorescence intensity in each nucleus in different samples, the values were normalized relative to the average of the untreated samples. The results showed that in the presence of psammaplin A, the acetylation levels of H3K9, H3K14, H3K27, H4K5, H4K8 and H4K12 were slightly increased at 100 nM and greatly increased at 1000 nM, similar to SAHA. The potency of TSA appeared to be roughly tenfold higher than that of psammaplin A and SAHA, and was effective at 10 nM. These results suggest that psammaplin A has a broad inhibitory spectrum, like TSA and SAHA.

## DISCUSSION

Here, we present a simple method for conducting a systematic analysis of the global level of multiple histone modifications in single cells. Using antibodies that were directly labeled with different fluorescent dyes, multiple modifications (mono-, di-, and trimethylation on a specific residue) were visualized using a wide-field fluorescence microscope. Image analysis with quantitation revealed the relative abundance of these modifications in hundreds of cells.

We have shown that the assay system for quantifying histone modification levels is considerably robust. First, various antibody concentrations in the range of 0.25-4 μg/ml did not affect the quantitative results, suggesting that optimization of staining conditions may not be crucial. Second, effects of antibody steric hindrance were not observed in the antibody combinations tested, assuring reliability of the multiplex analysis. In principle, further multiplexing is possible using sophisticated fluorescence microscopy techniques such as linear unmixing (Tsurui et al., 2000; Garbacik et al., 2018) and fluorescence lifetime imaging (Niehörster et al., 2016). Furthermore, multiplexed antibody staining could be used in mass cytometry imaging (Chang et al., 2017) to detect tens of different histone modifications simultaneously if the antibodies used remain functional after conjugation with metal-chelating polymers. In these higher multiplex systems, steric hindrance by many antibodies on chromatin could become problematic and so careful control experiments should be considered. Third, subtle fluctuations of nuclear focal planes among cells did not significantly affect the quantitative results when using a low NA objective lens. The cells used in this study were monolayers at subconfluent conditions and their nuclear focal planes were not significantly altered. Once the focus is set using the autofocus system, many nuclei in multiple fields are within the focus, and a range of objective lenses with NA 0.75-1.25 could be used for quantitative analysis. We used a NA 0.95 40× dry objective lens for most experiments in this study because dry lenses are convenient to use and the optical resolution and tolerance to focal shifts were adequate. For imaging at a higher resolution with quantitative analysis, a higher NA water-immersion lens can be used, but not a NA 1.4 100× oil immersion lens. For cells with relatively large variation in the focal plane, using an NA 0.75 20× dry objective lens may be more appropriate because the quantitative data for 2.4 μm out-of-focus images are almost the same as those of in-focus images. In the single-plane imaging used in this study, however, the analysis of mitotic cells is not straightforward because the focal planes of condensed chromosomes are different from those of interphase nuclei. Moreover, mitotic phosphorylation near the methylation or acetylation site interferes with the binding of many modification-specific antibodies.

We used an assay system to analyze changes in histone modifications during the cell cycle in response to KDM overexpression and inhibitor screening. The results of cell cycle dynamics and KDM target analyses were consistent with previous observations, thus confirming the validity of this method. Chemical screening, which resulted in the isolation of a known compound, also provided a proof of principle, suggesting that this method could be used for large-scale screening. Using manual handling, several 96-well plates could be processed within a few days; thus, analysis using an automated handling system would be more rapid and allow high throughput. This kind of application is particularly important because a chemical that regulates the epigenome could potentially serve as a drug target.

In this study, we analyzed histone modification dynamics using multicolor immunofluorescence-based single cell analysis. The experimental setup and quantitative analysis are not limited to histone modifications but can also be used for any nuclear proteins, such as HaloTag-tagged KDMs. Thus, this analytical system will be useful for future studies on nuclear protein and modification dynamics in response to stimuli and during the cell cycle.

## MATERIALS AND METHODS

### Generation, selection and purification of monoclonal antibodies

To generate monoclonal antibodies directed against H3K9un, H3K27un, H3K27me1 and H3K27me2, mice were immunized with synthetic peptides (H3K9un, ARTKQTARKSTGGKAPRKC; H3K27un, KQLATKAARKSAPATGGVKC; H3K27me1, KQLATKAAR(me1-K)SAPATGGVKC; and H3K27me2, KQLATKAAR(me2-K)SAPATGGVKC) coupled to the keyhole limpet hemocyanin, as described previously (Hayashi-Takanaka et al., 2015; Kimura et al., 2008). After generating hybridomas, clones were screened by enzyme-linked immunosorbent assay (ELISA) using the above-mentioned and following peptides: H3K9me1, ARTKQTAR(me1-K)STGGKAPRKC; H3K9me2, ARTKQTAR(me2-K)STGGKAPRKC; H3K9me3, ARTKQTAR(me3-K)STGGKAPRKC; H3K9ac, ARTKQTAR(ac-K)STGGKAPRKC; H3K27me3, KQLATKAAR(me3-K)SAPATGGVKC; and H3K27ac, KQLATKAAR(ac-K)SAPATGGVKC. The specificity of the selected clones was validated by ELISA and MODified Histone Peptide Array (Active Motif) (Fig. S3).

To conduct experiments using mice, all institutional and national guidelines for the care and use of laboratory animals were followed. All animal care and experimental procedures in this study were approved by the Hokkaido University Animal Experiment Committee (approval number 11-0109) and carried out according to the guidelines for animal experimentation at Hokkaido University, which houses the Mab Institute Inc. Animals were housed in a specific pathogen-free facility at Hokkaido University. Humane euthanasia of mice was performed by cervical dislocation by skilled personnel with a high degree of technical proficiency.

Antibodies against other histone modifications used in this study have been described previously (Hayashi-Takanaka et al., 2009, 2011, 2015; Kimura et al., 2008; Rechtsteiner et al., 2010). Antibody purification and fluorescence labeling were performed as described previously (Hayashi-Takanaka et al., 2011, 2014). Rabbit anti-HaloTag antibody (G9218) was purchased from Promega, and donkey anti-rabbit IgG (minimal cross-reaction to bovine, chicken, goat, guinea pig, Syrian hamster, horse, human, mouse, rat and sheep serum proteins; 711-005-152) was purchased from Jackson ImmunoResearch. To label anti-rabbit IgG with Alexa Fluor 750, 100 μg of anti-rabbit IgG was incubated with 8 μg of Alexa Fluor 750 *N*-succinimidyl ester (Thermo Fisher Scientific) in 100 mM NaHCO_3_ (pH 8.3) in a 100 μl reaction volume for 1 h with rotation using a rotator (Titec RT-60; 10 cm radius; ∼10 rpm). The labeled antibody was separated from free Alexa Fluor 750 using a PD-mini G-25 desalting column (GE Healthcare) pre-equilibrated with PBS (Takara). After addition of the reaction mixture (100 μl) on to the column resin, 0.45 ml of PBS was added and the flow-through discarded. The Alexa Fluor 750-conjugated antibody was eluted using 0.5 ml of PBS and concentrated up to ∼1 mg/ml using an Ultrafree 0.5 filter (10 k-cut off; Millipore).

### Cell culture

HeLa and hTERT-RPE1 cells have been described previously (Hayashi-Takanaka et al., 2015; Hayashi-Takanaka et al., 2019); 293T cells were obtained from Dr Kei Fujinaga (Sapporo Medical School) in 1991. Cells were grown in Dulbecco's modified Eagle's medium, high glucose (Nacalai Tesque) supplemented with glutamine (2 mM), penicillin (100 U/ml), streptomycin (100 μg/ml; Sigma-Aldrich) and 10% fetal calf serum (Gibco; Thermo Fisher Scientific).

### Immunofluorescence and microscopy

Cells were plated in a 12-well plate containing a coverslip (15 mm diameter; no. 1S; Matsunami). After more than 24 h of culture, the cells were fixed for 5 min with 4% paraformaldehyde in 250 mM HEPES (pH 7.4) containing 0.1% Triton X-100, permeabilized with 1% Triton X-100 in PBS for 20 min and blocked with Blocking One-P (Nacalai Tesque) for 15 min, as described previously (Kimura et al., 2008). The fixed cells were then incubated with labeled antibodies (0.2-1 μg/ml) and 0.1 μg/ml of Hoechst 33342 for 2 h at room temperature, and then washed three times with PBS for a total of 30 min. The cells in Fig. S2A were treated with 500 μl RNase A (100 μg/ml in PBS; Nacalai Tesque) overnight at 4°C, washed with PBS and incubated with 50 μl propidium iodide (20 μg/ml; Sigma-Aldrich) and labeled antibodies in PBS for 2 h. Coverslips were mounted on glass slides in Prolong Gold (Thermo Fisher Scientific) (Kimura et al., 2008). Fluorescence images were collected using a Ti-E inverted microscope (Nikon), under the operation of NIS Elements version 3.0 (Nikon), with a PlanApo VC or Lambda 40× dry (NA 0.95) objective lens equipped with an electron-multiplying charge-coupled device (EM-CCD; iXon+; Andor; normal mode; gain ×5.1) with filter sets (DAPI-1160A for Hoechst 33342, LF488-A for Alexa Fluor 488, LF561-A for Cy3, Cy5-4040A for Cy5, and FF01-732/68, FF757-Di01, FF01-776/LP for Alexa Fluor 750; Semrock). The exposure period was set at 100-1000 ms using a 75 W Xenon lamp as light source. Multipoint images were collected using a motorized X-Y stage with an autofocus system to retain a constant offset distance from the surface of the coverslip (Nikon Ti-E). The images in Fig. 2B and Fig. 3 were collected using PlanApo VC 20× dry (NA 0.75), Apo Lambda S 40× water-immersion (NA 1.25), PlanApo 60× water-immersion (NA 1.20), and PlanApo VC 100× oil-immersion (NA 1.4) objective lenses.

To identify cells in the S phase, cells were incubated in 10 μM EdU (Thermo Fisher Scientific) for 7.5 min and then fixed in 4% paraformaldehyde dissolved in 250 mM HEPES and 0.1% Triton X-100. The signal was detected using a Click-iT EdU Imaging Kit with Alexa Fluor 647 azide (Thermo Fisher Scientific).

### Image analysis

Fluorescence intensities of nuclei were measured using NIS Elements version 3.0 (Nikon). Background intensity outside the cells was subtracted from the images, and the area of nuclei in individual cells was determined using an automatic threshold of Hoechst signals. After visual inspection to fine-tune the threshold level in a single field (as shown in Fig. 1C), the same setting was applied to all other fields in the same sample. Proper nuclear segmentation was confirmed by visual inspection. A nuclear doublet was often segmented as a single nuclear area; in this case, each nucleus was separated using the ‘Separate objects manually’ tool in NIS Elements. Nuclei exhibiting abnormal shape and intensity, like those in apoptotic cells, were manually omitted using the ‘Delete object’ tool. The total intensity (average intensity×nuclear area) of each nucleus was measured for all fluorescence channels. When multiple fields are imaged using the autofocus system in a small area (within a few millimeters), both the background and signal intensities were similar enough among the images in different fields that no normalization was required for multipoint images in the same sample. To quantify approximately 500 cells, it takes approximately 10-40 min to segment nuclei in 25-35 fields with a 40× objective lens using a semi-manual system. Cells in the M phase were not included in this analysis because the area of mitotic condensed chromosomes differed substantially from that of interphase nuclei.

For plotting the intensity distribution, the Hoechst signal intensity in each nucleus was normalized using the fifth lowest and fifth highest intensity nuclei set as 1 and 2, respectively (because the lowest and highest intensity nuclei were sometimes outliers). For histone modification, the intensity in each nucleus was normalized using the average. The normalized intensities were plotted on a linear scale for cell cycle analysis and on a log scale for perturbation assays, such as KDM expression and HDAC inhibitor treatments, using Mathematica version 9-11 (Wolfram Research). The source data and Mathematica code for making plots and heatmaps (Fig. 1D-F) are available at https://github.com/YTHayashi/SingleCellAnalysis.

### Flow cytometry

HeLa cells grown on a 10 cm dish (2.4×10^6^) were trypsinized, collected via centrifugation (1300×***g*** for 3 min), and resuspended in 300 μl of PBS. The cells were fixed by adding and mixing with 700 μl of 100% ethanol, resulting in a 70% ethanol solution, and stored for 1 h at −20°C. After centrifugation (1300×***g*** for 3 min at 4°C), the 70% ethanol was discarded and the cells resuspended in 1 ml of PBS. The cells were then washed twice with PBS and incubated in 0.5 ml Blocking One-P (Nacalai Tesque) for 15 min. Subsequently, the cells were incubated with three labeled antibodies (0.2-1 μg/ml) and Hoechst 33342 (0.1 μg/ml) in a 500 μl reaction mixture for 2 h at room temperature. After washing with PBS, the cells were analyzed using a FACSAria IIIu (BD Biosciences) featuring 375, 488, 561 and 633 nm lasers. The cells stained with single antibodies and Hoechst 33342 were used to adjust the compensation parameters.

### HaloTag-KDM expression

The following Kazusa cDNA clones were used to express the HaloTag-KDM expression vectors: KDM1A (FHC 00571), KDM2A (FHC 00712), KDM3A (FHC 01605), KDM3B (FHC 05559), KDM3C (FHC36094E), KDM4A (FHC00602), KDM4B (FHC00669), KDM4C (FHC00635), KDM4D (FHC06842), KDM5A (FHC01704), KDM5B (FHC27753), KDM5C (FHC11536), KDM5D (FHC00039) and KDM6B (FHC00327). To conduct transient expression assays, 293T cells were plated in a 12-well plate containing a coverslip in each well, as described above, at 20-30% confluency. On the next day, transfection was performed using GeneJuice transfection reagent (Merck), according to the manufacturer's instructions; briefly, 0.5 μg of DNA was mixed with 50 μl of Opti-MEM (Thermo Fisher Scientific) and then 1.5 μl of GeneJuice Reagent was added to the mixture. Approximately 24 h after transfection, the cells were fixed, permeabilized and blocked, as described above. Cells were stained with rabbit anti-HaloTag antibody (1:1000) for 2 h and then with Alexa Fluor 750-labeled anti-rabbit IgG (2 μg/ml; secondary antibody), dye-labeled histone modification-specific antibodies and Hoechst 33342 at room temperature.

### Determination of HDAC inhibitory activity in marine organism extract

To screen chemicals that affect histone modification levels, MDA-MB-231 cells were cultured in 96-well glass-bottom plates (AGC techno glass) in DMEM containing 10 µg/ml extracts (2×10^3^ to 4×10^3^ cells in 100 µl medium in each well), incubated in a CO_2_ incubator for 18-24 h, and fixed and stained as described above. To examine the effect of HDAC inhibitors on histone acetylation levels, cells were incubated for 4 h in the presence of inhibitors prior to fixation. Fluorescence images of individual wells were collected using an inverted microscope (Ti-E; Nikon) equipped with an X-Y stage (Nikon), using a PlanApo 20× dry objective lens (NA 0.75).

The marine sponge sample (S09420) that enhanced the levels of H3K9ac and H3K27ac was extracted from a sponge identified as *Aplysilla* sp., which was collected from Pisira Island, Chuuk, Federated States of Micronesia (7°29.04'N, E 151°49.69'E) in September 2009. The material was stored at −30°C until required for analysis. The frozen material (300 g) was extracted five times with methanol (1000 ml) at room temperature. The crude extract was concentrated and partitioned into aqueous and chloroform layers. The aqueous layer was extracted with 1-butanol and combined with the chloroform layer. The organic layer was subjected to a modified Kupchan method (Kupchan et al., 1973) yielding 1-hexane, chloroform and aqueous methanol layers. The chloroform layer was applied to ODS flash chromatography (ODS-A 120-S150; YMC) and the column was eluted in a stepwise manner with methanol:water (5:5 and 7:3), acetonitrile:water (7:3 and 17:3), methanol and chloroform:methanol:water (6:4:1). The methanol:water (7:3) fraction, which contained the activity to increase H3K9ac and H3K27ac levels, was further separated by reversed-phase HPLC (COSMOSIL 5C_18_ AR-II) in 40% acetonitrile to yield pure psammaplin A as the active substance (255 mg). The structure of psammaplin A was identified by comparison of the NMR spectra and MS data with a previous study (Arabshahi and Schmitz, 1987).

## Supplementary Material

Supplementary information

Reviewer comments
