# Supplementary material for: Histone modification dynamics as revealed by multicolor immunofluorescence-based single-cell analysis
Source: J Cell Sci. 2020 Jul 21;133(14):jcs243444. doi: 10.1242/jcs.243444 (PMC7390643; doi:10.1242/jcs.243444)
Supplement: Supplementary information [file joces-133-243444-s1.pdf]

Histone modification dynamics as revealed by a multicolor immunofluorescence-based single-cell analysis

Yoko Hayashi-Takanaka[1,2,*], Yuto Kina[3], Fumiaki Nakamura[3], Leontine E. Becking[4], Yoichi Nakao[3], Takahiro Nagase[5], Naohito Nozaki[6] and Hiroshi Kimura[1,7,*]

Supplementary Information (Figure S1-S6)

Journal of Cell Science • Supplementary information

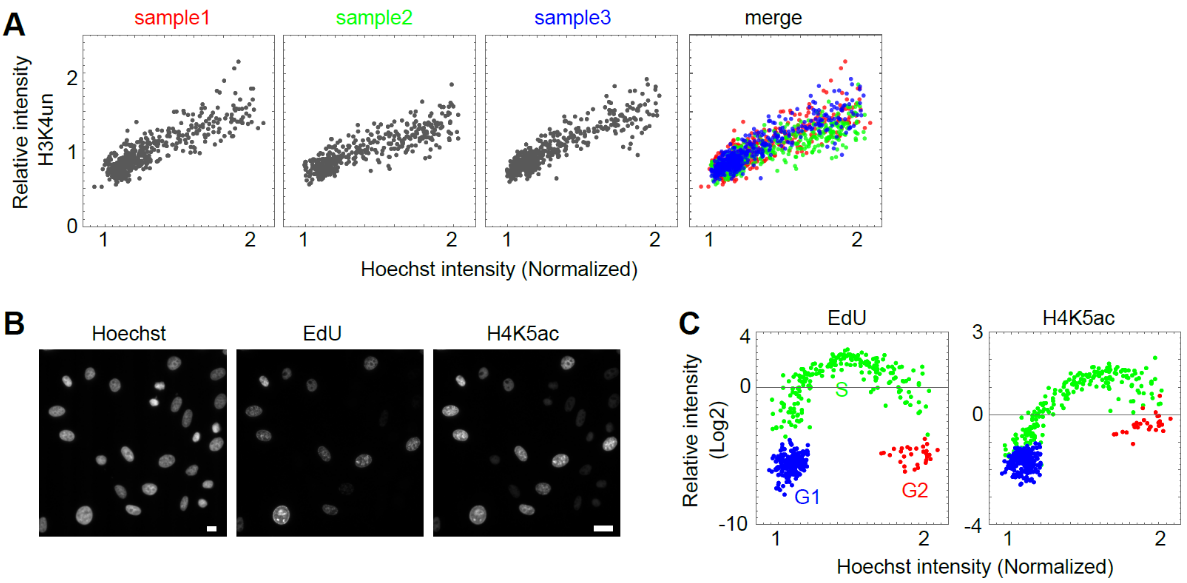

Figure S1. Reproducibility of dot plot analysis and identification of cells in the S phase. A. The levels of H3K4un during the cell cycle in independent experiments. Three biological independent replications were performed (indicated in red, green, and blue), as in Fig. 1. The results of all three experiments were merged to examine the differences in the distribution among individual experiments. The sample1 is a reproduction of the data in Figure 1 (Cell number = 450). Cell number = 400 (sample 2) and 500 (sample 3). **B** and **C**. Level of H4K5ac, a marker for S-phase cells. To confirm that higher H4K5ac level can be an indication of the S phase (Hayashi-Takanaka et al., 2015), HeLa cells were pulse-labeled using EdU for 7.5 min to label replicated DNA. **B**. Cells fixed and stained with H4K5ac-specific antibody (Alexa Fluor 488), Alexa Fluor 647-azide (to label EdU), and Hoechst 33342. **C**. The signal intensity of EdU or H4K5ac. The relative intensities of both EdU and H4K5ac are shown on a log scale because EdU signals exhibited a high dynamic range in the intensity. EdU signals were clearly higher in the S phase than in the G1 and G2 phases (distinguished by the DNA content), and H4K5ac signals showed a pattern similar to EdU, exhibiting higher levels in the S phase (green). Although H4K5ac level was higher in G2 cells (red) than in G1 cells (blue), the G2 fraction could be separated based on Hoechst signals. Cell number = 450.

Journal of Cell Science • Supplementary information

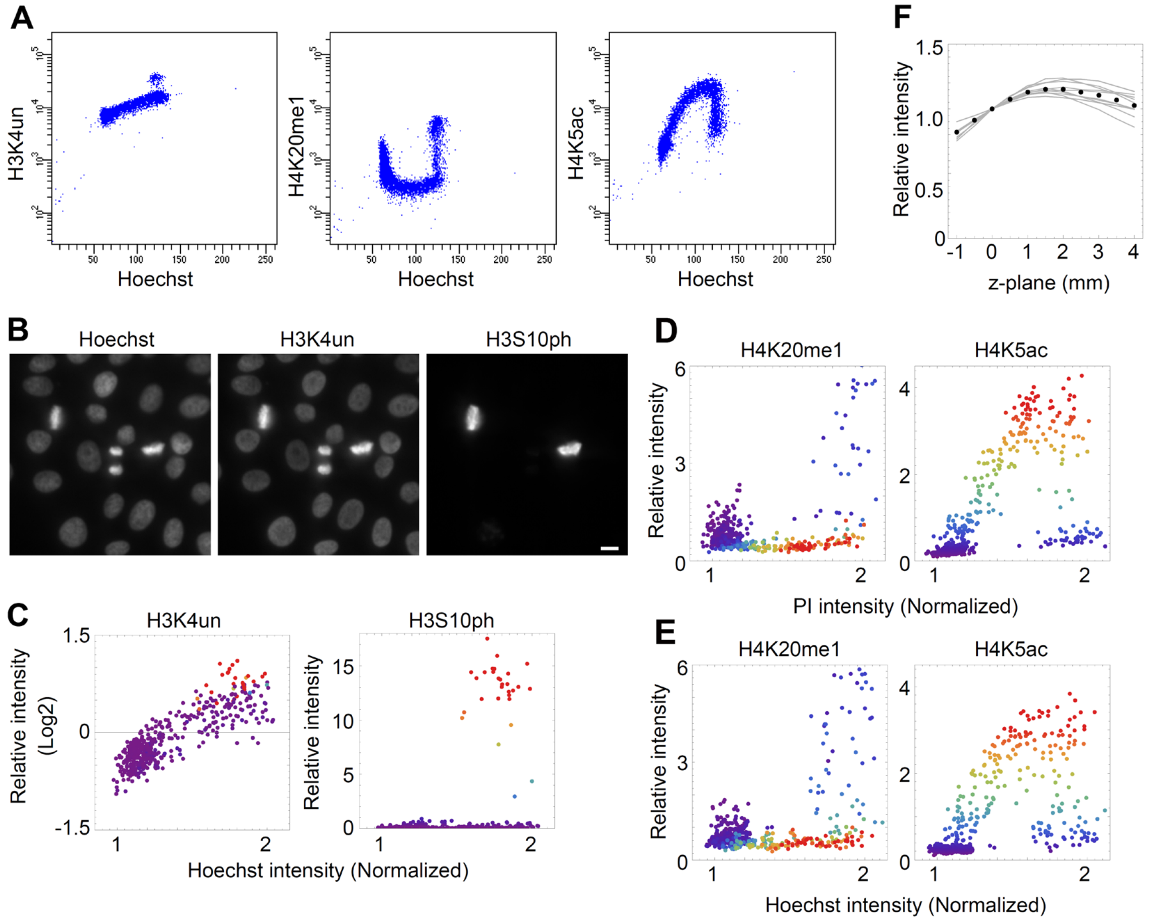

Figure S2. Comparison between flow cytometry and microscopy profiles.
A. Fluorescence-activated cell sorting (FACS) profiles. HeLa cells were stained using Hoechst and antibodies specific for H3K4un (Alexa Fluor 488), H4K20me1 (Cy3), and H4K5ac (Cy5). The parameters for crosstalk compensation were adjusted using cells stained with Hoechst or a single antibody. The histone signals were plotted on a log scale, as the dynamic ranges were high in H4K20me1 and H4K5ac. The profiles are similar to those obtained using microscopy, as shown in Figs. 1 and S1, with the exception of the presence of a cluster with the highest H3K4un and high Hoechst signals. This cluster appears to be derived from mitotic cells as observed using the microscopic analysis below. **B** and **C**. HeLa cells stained with antibodies specific for H3K4un (Alexa Fluor 488) and mitosis-specific phosphorylated H3S10 (H3S10ph; Cy3). **B**. Typical microscopic images. Scale bar = 10 µm. **C**. Plots. Mitotic cells associated with intense H3S10ph signals (indicated in red/orange color) show high levels of H3K4un, but not as high as those by FACS, and also show moderately high (not the highest) Hoechst intensities. The lower signals of H3K4un and Hoechst than those of FACS are due to the smaller areas of mitotic chromosomes than nuclear areas in a single focal plane by microscopy, which is contrasting to the detection of total fluorescence by FACS. **D** and **E**. Comparison of DNA staining with propidium iodide (PI) and Hoechst. Fixed and permeabilized HeLa cells were stained with antibodies specific for H4K20me1 (Alexa Fluor 488) and H4K5ac (Cy5) and either PI with RNase (**D**) or Hoechst (**E**). Profiles are similar between PI- and Hoechst-stained samples. Cell numbers = 450. **F**. Hoechst intensities of mitotic chromosomes in different z-planes. The total intensity (average intensity × area) of Hoechst in mitotic chromosomes was measured in each plane, and relative values to that of the focal plane in interphase nuclei are plotted. The dots and lines indicate the averages of 12 cells and individual cell data, respectively. The planes 1.5–2 µm above the interphase nuclear focal plane are 1.15 times higher.

Journal of Cell Science • Supplementary information

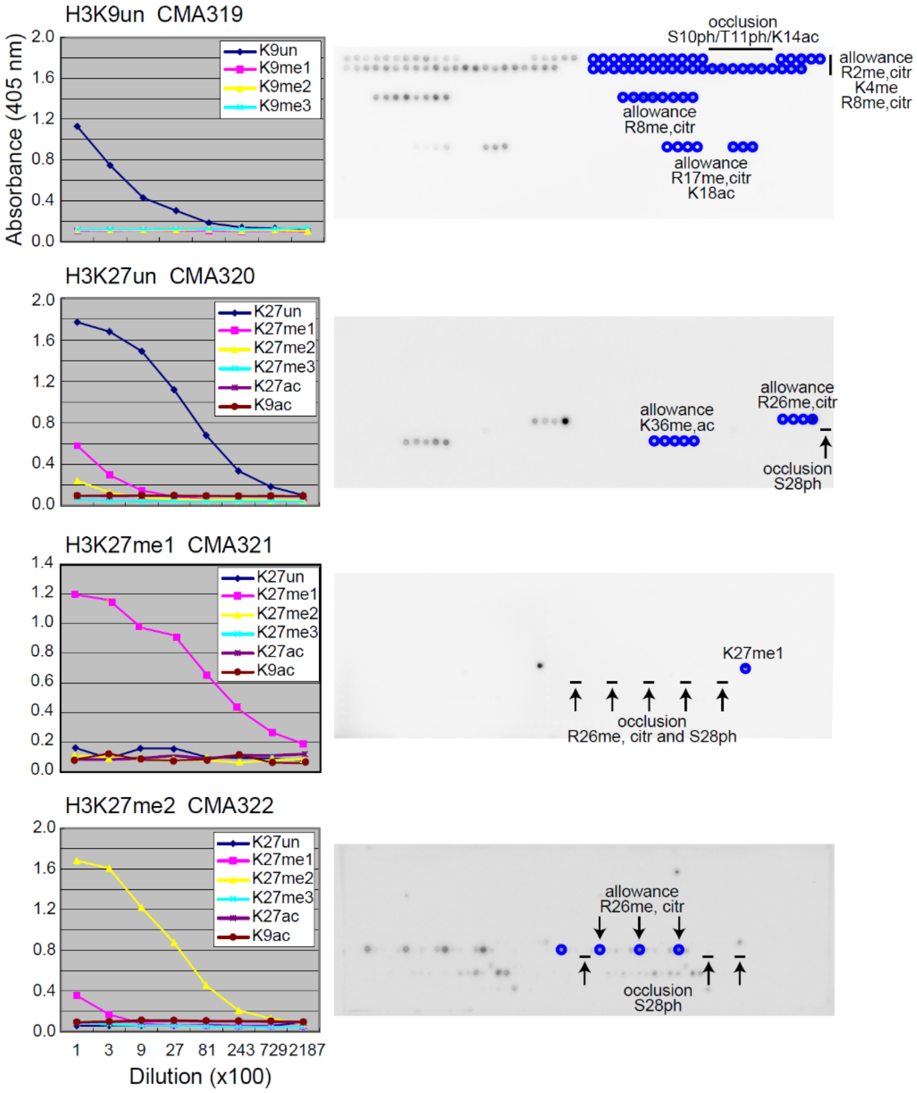

Figure S3. Characterization of new mouse monoclonal antibodies.
The left panel shows the evaluation of the specificity of monoclonal antibodies by ELISA. Microtiter plates coated with bovine serum albumin-conjugated peptides were incubated with 3-fold dilutions of each antibody, starting from a 1:100 dilution of a hybridoma culture supernatant. After incubation with peroxidase-conjugated secondary antibody and washing, the colorimetric signal of tetramethylbenzidine was detected by measuring the absorbance at 405 nm using a plate reader. Peptides that reacted with the individual antibodies are indicated. The right panel shows the evaluation of the specificity of the monoclonal antibodies and the effect of neighboring modifications using a histone peptide array. Positive spots are indicated in blue in the right duplicate. The effects of neighboring modifications on antibody binding (allowance and occlusion) are indicated. Clone CMA319 (H3K9un) bound to peptides harboring unmodified H3K9, regardless of the methylation or citrullination of R2, R8, or R17, methylation of K4, and acetylation of K18. CMA319 did not bind to the peptides with phosphorylation of S10 or T11 and acetylation of K14. The clone CMA320 bound to peptides containing unmodified K27, regardless of the methylation and citrullination of R26 or methylation and acetylation of K36, but its binding was prevented by the phosphorylation of S28. CMA321 bound to the peptide harboring K27me1, but its binding was prevented by the methylation and citrullination of R8 and phosphorylation of S28. CMA322 bound to the peptides harboring K27me2, regardless of the methylation and citrullination of R26, but its binding was prevented by the phosphorylation of S28. me, methylation; citr, citrullination; ac, acetylation; ph, phosphorylation.

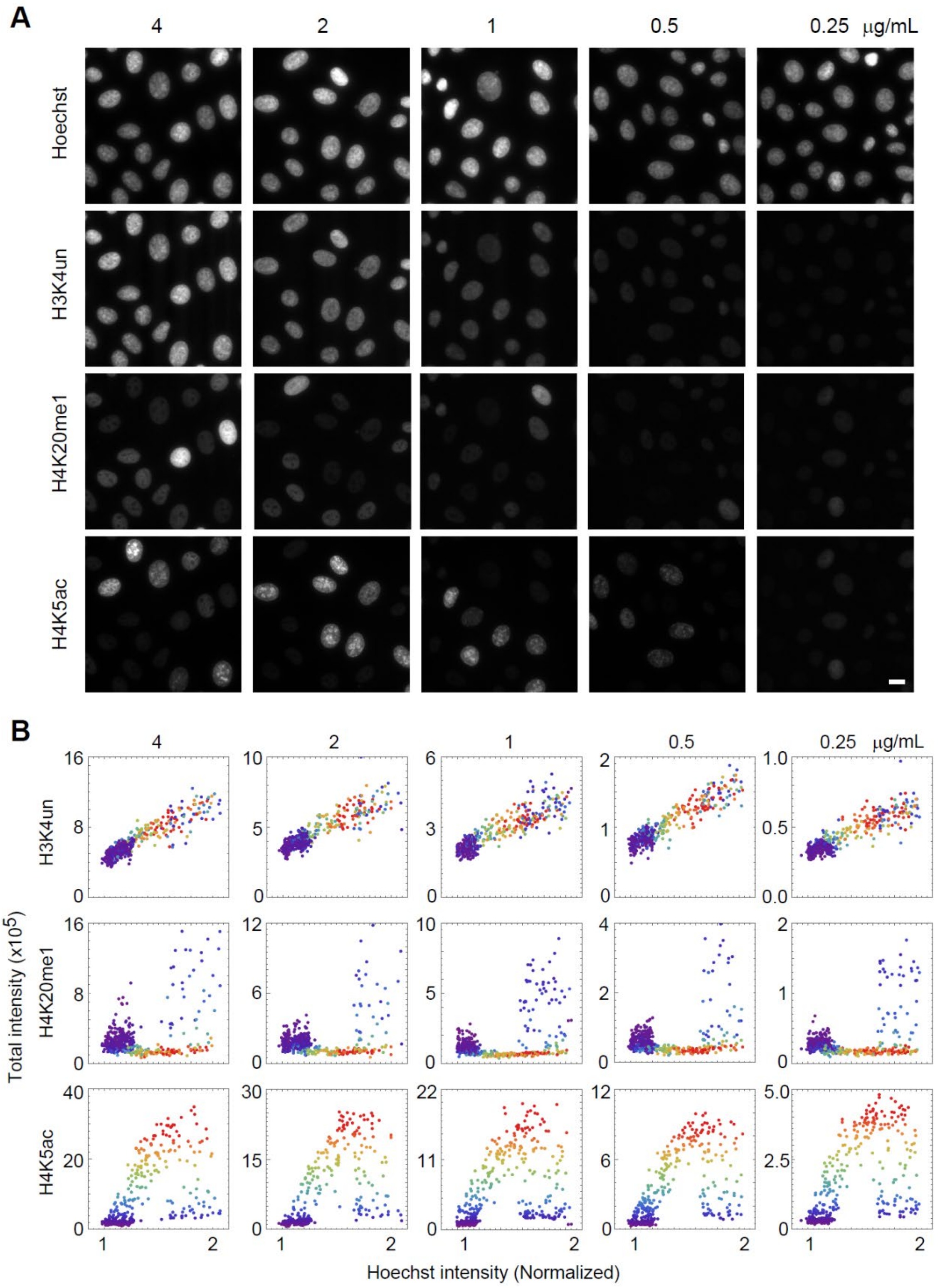

Figure S4. Effects of antibody concentration on the intensity profiles.
Fixed and permeabilized HeLa cells were stained with various concentrations of antibodies specific for H3K4un (Alexa Fluor 488), H4K20me1 (Cy3), and H4K5ac (Cy5), and a constant concentration of Hoechst33342. A. Fluorescence images. The same microscope settings were used for all samples and the contrast was unchanged. B. Intensity profiles. Total intensities (mean intensity × nuclear area) of histone modification and normalized Hoechst intensities are plotted with the H4K5ac level in rainbow color. Scale bar = 10 μm. Cell numbers = 450.

Journal of Cell Science • Supplementary information

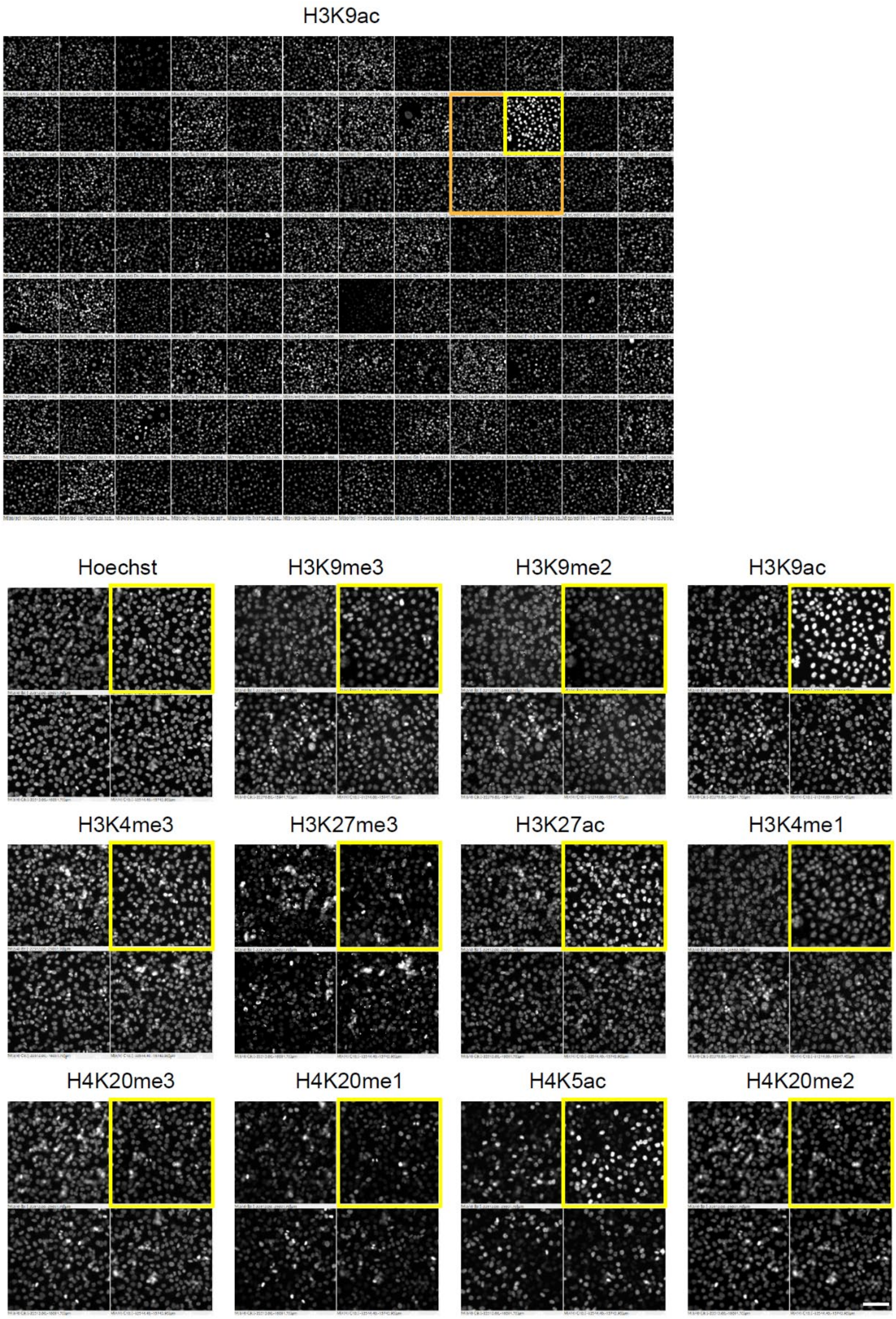

Figure S5. Screening of marine organism extracts based on changes in histone modification levels.
For the set shown in the orange frame of H3K9ac, the results stained with Hoechst and other histone modifications are also shown. The yellow frame shows cells treated with Psammaplin A. Higher levels of acetylation (H3K9ac and H3K27ac) and lower levels of methylation (H3K9me3, H3K9me2, and H4K20me1) are observed. Scale bars = 100 μm.

Journal of Cell Science • Supplementary information

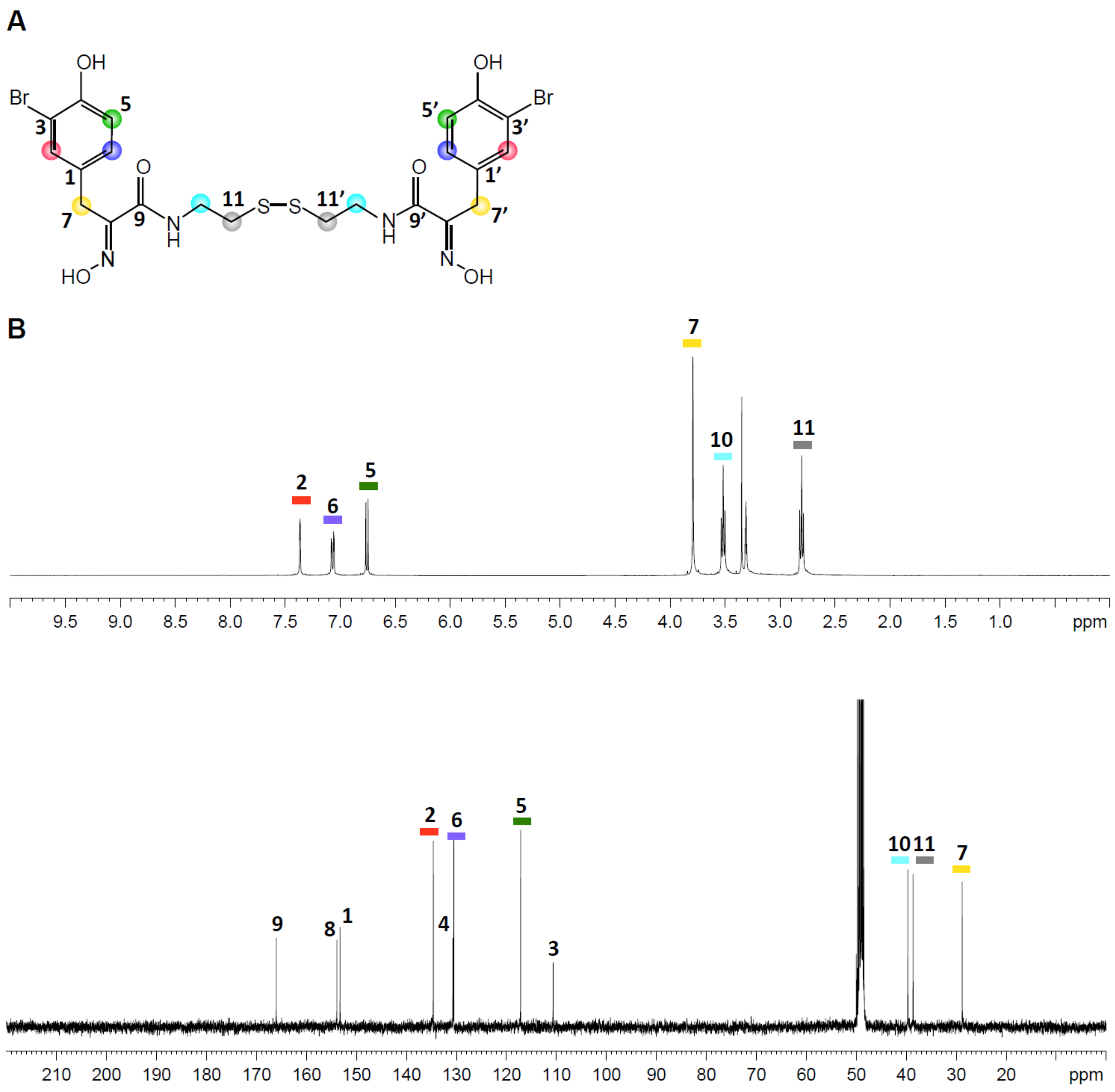

Figure S6. NMR spectrum of fractions containing psammaplin A.
A. Structure of psammaplin A. B. 1H NMR spectrum (CD3OD, 400 MHz) of purified psammaplin A. NMR signals corresponding to protons in the structures are numbered.
