# Supplementary material for: Histone modification dynamics as revealed by multicolor immunofluorescence-based single-cell analysis
Source: J Cell Sci. 2020 Jul 21;133(14):jcs243444. doi: 10.1242/jcs.243444 (PMC7390643; doi:10.1242/jcs.243444)
Supplement: Reviewer comments [file joces-133-243444_review_history.pdf]

Journal of Cell Science

Histone modification dynamics as revealed by multicolor immunofluorescence-based single-cell analysis

Yoko Hayashi-Takanaka, Yuto Kina, Fumiaki Nakamura, Leontine E. Becking, Yoichi Nakao, Takahiro Nagase, Naohito Nozaki and Hiroshi Kimura
DOI: 10.1242/jcs.243444

Editor: Maria Carmo-Fonseca

Review timeline
Original submission: 2 January 2020
Editorial decision: 10 February 2020
First revision received: 4 May 2020
Accepted: 8 June 2020

Original submission

First decision letter

MS ID#: JOCES/2019/243444

MS TITLE: Histone modification dynamics as revealed by a multicolor immunofluorescence-based single-cell analysis

AUTHORS: Yoko Hayashi-Takanaka, Yuto Kina, Fumiaki Nakamura, Leontine E Becking, Yoichi Nakao, Takahiro Nagase, Naohito Nozaki, and Hiroshi Kimura
ARTICLE TYPE: Tools and Resources

We have now reached a decision on the above manuscript.

To see the reviewers' reports and a copy of this decision letter, please go to: https://submit-jcs.biologists.org and click on the 'Manuscripts with Decisions' queue in the Author Area. (Corresponding author only has access to reviews.)

As you will see, the reviewers raise a number of substantial criticisms that prevent me from accepting the paper at this stage. They suggest, however, that a revised version might prove acceptable, if you can address their concerns. If you think that you can deal satisfactorily with the criticisms on revision, I would be pleased to see a revised manuscript. We would then return it to the reviewers.

Please ensure that you clearly highlight all changes made in the revised manuscript. Please avoid using 'Tracked changes' in Word files as these are lost in PDF conversion.

I should be grateful if you would also provide a point-by-point response detailing how you have dealt with the points raised by the reviewers in the 'Response to Reviewers' box. Please attend to all of the reviewers' comments. If you do not agree with any of their criticisms or suggestions please explain clearly why this is so.

© 2020. Published by The Company of Biologists under the terms of the Creative Commons Attribution License (https://creativecommons.org/licenses/by/4.0/).

Reviewer 1

Advance summary and potential significance to field

This is the development of a simple multichannel fluorescence method for assessing cell cycle dynamics of histone posttranslational modifications. In some ways, the approach is not new. Quantifying signals from fluorescence images to assess the modification state of histones is essentially what high content analysis does routinely. That said, I think that this paper contributes in introducing a more accessible assay to investigators interested in histone posttranslational modifications) and I believe that it is suitable as a tools and techniques article.

Comments for the author

1. A major and unnecessary limitation of the study is that it does not incorporate the mitotic cell population. This is particularly relevant to the dynamics of lysine 9 di and trimethylation and lysine 27 trimethylation.
For example, the profiles obtained for lysine 9 me2 and me3 are very similar to what was reported by McManus et al. (JBC 281:8888-8897), which used similar methods but included mitotic cells. Here mitosis-specific dynamics were observed that would significantly change the interpretation of the cell cycle-dependent regulation of these modifications. Given that these cells are easy to independently segment and quantify, the inability to do this simultaneously with the interphase cells is not a valid reason for not incorporating them into the study. Indeed, mitosis-specific dynamics of histone acetylation are also prevalent and this assay would have considerably more value if it incorporated the entire cell cycle.
2. The normalization of the fluorescence of individual nuclei is not well described. I believe that this involved normalizing for average intensity per field using DAPI and then applied the same correction value for each cell in the field, with each channel treated identically. That is not clear in the description of the method or its presentation in the results.
3. Because this assay, if widely used, is likely to be employed by people without significant experience in the techniques, the authors should emphasize in the text of the results that they are employing a (relatively)
low numerical aperture objective and the importance of this for capturing the z-depth of the data. Do higher N.A. objectives produce more variable results because they don't collect the entire depth of the nucleus? What N.A. is acceptable for this analysis? This detail should be provided if the assay is to be successfully adopted in a widespread manner.
4. One control that should be done is demonstrating that the co-labeling does not suppress the signal (i.e., the binding of one antibody to the nucleosome does not limit the binding of a second such that the observed signal is sensitive to what it is co-stained with).
5. The sensitivity of the newly generated antibodies to PTMs outside of the amino acid of interest, illustrated in supplementary Figure 5, should be clearly noted when discussing the interpretation of the affected modification.
6. In this study, they have multiplexed up to 4-channels. With spectral deconvolution methods, fluorescence lifetime instruments, etc..., there is opportunity to observe even larger numbers of PTMs in the same cell simultaneously. It would be useful to incorporate into the manuscript, perhaps in the discussion, a discussion of the potential to go beyond what was done here. It would also be useful to incorporate into the discussion what they know about competition between antibodies when different nucleosomal binding sites are employed. Presumably, at some point, steric hindrance would arise between antibodies as one increased the number of PTMs that one examines simultaneously.
7. Page 7-8: "Since H3K9me1 is also enriched in the gene body of transcribed genes, chromatin containing this modification is likely to be replicated during the early S phase, and it can be restored during the S phase even the G9a/GLP complex is the responsible methyltransferase (Lin et al., 2016) as H3K9me2, which is restored after the S phase (see below) (Alabert et al., 2015)." The statement "even the G9a/GLP complex is the responsible"--there is a word or something missing from this statement and it currently does not make sense.

© 2020. Published by The Company of Biologists under the terms of the Creative Commons Attribution License (https://creativecommons.org/licenses/by/4.0/).

Reviewer 2

Advance summary and potential significance to field

This manuscript presented a method using multi-color immunofluorescence together with Hoechst staining to analyze a large set of histone modifications in cell cycle. Then the authors demonstrated the use of this assay to screen the extracts of marine organisms and identified a compound that inhibits histone deacetylase. The method itself is not innovative, because such measurement of fluorescence intensity to quantify protein/DNA content is often used in biological studies simply as an established method. What is valuable here is the extensive analysis for the correlation between a large number of histone marks and DNA content to characterize their dependence on cell cycle. If the analysis was done automatically and the authors share their tools, it could be useful for the scientific community. However, the authors provided little details on the technical aspects of the tool. Therefore, if the authors address all of the technical concerns and enhance the technical contents for their analysis, this work can be published.

Comments for the author

(1) The authors used fixed cells stained Hoechst and measure the intensity level as a measure of DNA content, which is then correlated with the fluorescence intensity from different histone marks to measure the “dynamic” changes of histone marks during the cell cycle. This is the underlying basis of the method presented in this paper. This basis is correct only when the variation of the measured Hoechst intensity is due to the different cell cycle phases. However, as the authors used the objective with NA = 0.95, the depth of focus is only in the range of 1-2 microns. HeLa cells can be ~5-10um thick. Therefore, the effect of focal position should be carefully studied. For example, at a given focal plane, how many cells are in focus and how many cells are out of focus? How do authors distinguish “in-focus” from “out-of-focus” cells in an automatic fashion? Further, the authors need some validation step to confirm that their method of measuring DNA content (in terms of intensity distribution) with flow cytometry that is the gold standard in quantifying the DNA content.
(2) The authors presented Hoechst fluorescence intensity in a linear scale, but the intensity from histone marks in a logarithmic scale. It is not clear why they want to present the data this way, as these two are not power-law dependence.
(3) I wonder whether the results remain robust if another DNA binding dye is used. Hoechst 33342 has a preference to bind adenine-thymine (A-T) regions of DNA. Validation for at least Figure 1 or 2 using another dye will further validate their findings.
(4) The authors did show the repeatability from 3 replicates. What is the impact of the various antibody efficiency?
(5) There are a few technical components missing in this paper. Given this is presented as a method paper, the throughput of this method needs to be discussed. Further, cell nuclei segmentation is an important step in the image analysis. More detailed should be discussed. Is it fully automated or based on manual selection?

Reviewer 3

Advance summary and potential significance to field

The work contributes an important robust method, which can be used in any epigenetic laboratory. It also opens a source of directly labeled antibodies to histone modifications, which is an extremely valuable addition to epigenetic arsenal.

Comments for the author

The paper by Hayashi-Takanaka et al presents and discusses a robust method of screening post-translational histone modifications in populations of cultured cells based on a single cell analysis. In this and their previous paper, the authors generated a whole library of modification-specific antibodies directly conjugated to fluorochromes. After performing standard immunostaining by combinations of various antibodies, cells were imaged by epifluorescence microscope. Then automated nuclear segmentation and measuring of total nuclear fluorescence intensities were

© 2020. Published by The Company of Biologists under the terms of the Creative Commons Attribution License (https://creativecommons.org/licenses/by/4.0/).

performed using non-dedicated software, which can be substituted by any other image-analysis programs, including ImageJ plugings. The method does not require sophisticated equipment; simplicity and robustness of the method allows quick and reliable acquisition and analysis of thousands of nuclei of cultured cells.

To demonstrate efficacy of their approach, the authors monitored changes in 21 histone modifications over the cell cycle. They propose a clever way to estimate the cell cycle stage by combination of DNA content measurement (based on counterstaining dye) and immunostaining of a marker modification enriched in S-phase (H4K5ac). By this way, the authors demonstrated a clear differential dynamics of histone modifications, in particular, increasing of “active” modifications during S-phase and “inactive” modifications towards G2 continuing into G1 of the next cell cycle.

The second application was investigation of targets of lysine demethylases (KDMs). For this, cultured cells were transiently transfected with one of the 14 HaloTag-KDMs and then, by applying their microscopy-based screening method, the authors systematically tested changes in methylation levels. This way the authors figured out with impressive clarity which methylation sites of H3K4,K9,K27,K36 and H4K20 the mono-, di- or tri-methylation was increased. In addition, using the same approach, the authors have tested chemical compounds extracted from a marine sponge and searched for extract fractions that affect histone modifications. As result, they identified two fractions containing psammaplin A, a chemical inhibiting histone deacetilases (HDACs), and further characterized its properties by comparing with other known HDAC inhibitors such as TSA and SAHA.

All-in-all, I find this work very useful as it contributes an important robust method, which can be used in any epigenetic laboratory. It also opens a source of directly labeled antibodies to histone modifications, which is an extremely valuable addition to epigenetic arsenal. Therefore, I do recommend this manuscript for publication.

I have only minor comments to the text and figures of the paper:

(1) Although the presented method is based on a high throughput imaging and thus employs small objective lense (40x), to demonstrate a quality of immunostainings, images of at least several histone modifications have to be shown at a higher modification – with at least 63x and preferably as optical mid-nuclear sections. This should allow to see differential distribution of different modifications and thus to prove specificity and quality of immunostaining

(2) Figure 1F: for clarity, direction from G2 to G1 should be added to the heat map on the right

(3) Classification of modifications into three groups seems to me somewhat subjective: e.g., why H4K16ac belong to the second and not to the first group?

(4) Figure 2C: the plot looks very busy and difficult to read; it would be more clear if the three groups of modifications are color-coded and the font of the lettering is reduced

(5) Fig.3C: markings “n=400” and “top50” have to be added to columns, as they stand in 3B.

(6) M&M: throughout the paper, the authors indicate Hochst as the nuclear stain, however, in the description of the used microscope they indicate a filter for DAPI. What was used – DAPI or Hoechst?

First revision

<u>Author response to reviewers' comments</u>

We thank the reviewers for their constructive feedback. Based on their recommendations, we have added a new section entitled “Evaluating the robustness of the assay system” in the Results section along with new figures (Fig. 2, 3, S2, and S4), describing the effects of the antibody concentration, double staining (vs. single staining), and numerical aperture (NA) of the objective lens. These results have also been discussed in the Discussion. We have also made the necessary changes to address all the concerns raised. The dot plots are now rescaled to linear graphs

© 2020. Published by The Company of Biologists under the terms of the Creative Commons Attribution License (https://creativecommons.org/licenses/by/4.0/).

because most of the histone modifications fluctuate within a two-fold range during the cell cycle, as suggested by Reviewer 2.

Revisions in the manuscript are indicated by red text.

Reviewer 1 Comments for the Author:

1. A major and unnecessary limitation of the study is that it does not incorporate the mitotic cell population. This is particularly relevant to the dynamics of lysine 9 di and trimethylation and lysine 27 trimethylation. For example, the profiles obtained for lysine 9 me2 and me3 are very similar to what was reported by McManus et al. (JBC 281:8888- 8897), which used similar methods but included mitotic cells. Here mitosis-specific dynamics were observed that would significantly change the interpretation of the cell cycle-dependent regulation of these modifications. Given that these cells are easy to independently segment and quantify, the inability to do this simultaneously with the interphase cells is not a valid reason for not incorporating them into the study. Indeed, mitosis-specific dynamics of histone acetylation are also prevalent and this assay would have considerably more value if it incorporated the entire cell cycle.

Thank you for this important comment. We agree that the inclusion of mitotic cells would further support the assay's application. Unfortunately, the mitotic cells were excluded from most of assays for two reasons. First, the area of mitotic chromosomes in the nuclear focal plane is smaller than that of a nucleus; therefore, the total intensity is underestimated. This has been described in the Results section (p. 5) with new Fig. S2A- C, F and in Discussion section (p. 16). Second, most of the methyl- and acetyl-specific antibodies are sensitive to mitotic phosphorylation on a neighboring Ser/Thr residue; this has also been described in the Results (p. 5–6) and Discussion (p. 16) sections.

2. The normalization of the fluorescence of individual nuclei is not well described. I believe that this involved normalizing for average intensity per field using DAPI and then applied the same correction value for each cell in the field, with each channel treated identically. That is not clear in the description of the method or its presentation in the results.

We apologize for this ambiguity; the normalization methods have been described in detail (p. 21).

3. Because this assay, if widely used, is likely to be employed by people without significant experience in the techniques, the authors should emphasize in the text of the results that they are employing a (relatively) low numerical aperture objective and the importance of this for capturing the z-depth of the data. Do higher N.A. objectives produce more variable results because they don't collect the entire depth of the nucleus? What N.A. is acceptable for this analysis? This detail should be provided if the assay is to be successfully adopted in a widespread manner.

According to this suggestion, we performed analyses using several objective lenses with different NA and magnifications. When the focus is well adjusted to the equatorial section of nuclei using an autofocus system, not much difference in quantified data was observed in lenses with NA 0.75–1.25. However, the variation of Hoechst intensity broadened with the use of a 100× lens (NA 1.4) (new Fig. 2B,C). We also observed that a lens with decreased NA (20×; NA 0.75) was more tolerant to focal shift (new Fig. 3). These data have been included in the revised manuscript (p. 7–8).

4. One control that should be done is demonstrating that the co-labeling does not suppress the signal (i.e., the binding of one antibody to the nucleosome does not limit the binding of a second such that the observed signal is sensitive to what it is co-stained with).

We investigated whether the binding of one antibody to histones interferes with that of another by comparing single and double antibody staining profiles. No differences were observed in any of the tested combinations, probably because not all epitopes on nucleosomes are covered by an antibody. These results have been described in the manuscript (p. 6–7 and new Fig. 2A).

5. The sensitivity of the newly generated antibodies to PTMs outside of the amino acid of interest, illustrated in supplementary Figure 5, should be clearly noted when discussing the interpretation of the affected modification.

Based on this suggestion, we have described the properties of new antibodies in the primary text

© 2020. Published by The Company of Biologists under the terms of the Creative Commons Attribution License (https://creativecommons.org/licenses/by/4.0/).

together with the sensitivity to phosphorylation in previous antibodies (p 5– 6).

6. In this study, they have multiplexed up to 4-channels. With spectral deconvolution methods, fluorescence lifetime instruments, etc..., there is opportunity to observe even larger numbers of PTMs in the same cell simultaneously. It would be useful to incorporate into the manuscript, perhaps in the discussion, a discussion of the potential to go beyond what was done here. It would also be useful to incorporate into the discussion what they know about competition between antibodies when different nucleosomal binding sites are employed. Presumably, at some point, steric hindrance would arise between antibodies as one increased the number of PTMs that one examines simultaneously.

We have included a discussion on the possibility of expanding to more multiplexing noting the caution of considering steric hindrance from the antibodies (p 15–16). Although we have added new data supporting the minute effect of one antibody to another in double labeling (new Fig. 2A), studies with more controls are necessary for higher multiplexing.

7. Page 7-8: "Since H3K9me1 is also enriched in the gene body of transcribed genes, chromatin containing this modification is likely to be replicated during the early S phase, and it can be restored during the S phase even the G9a/GLP complex is the responsible methyltransferase (Lin et al., 2016) as H3K9me2, which is restored after the S phase (see below) (Alabert et al., 2015)." The statement "even the G9a/GLP complex is the responsible"--there is a word or something missing from this statement and it currently does not make sense.

This sentence has been modified for improved clarity (p. 10).

Reviewer 2 Comments for the Author:
(1) The authors used fixed cells stained Hoechst and measure the intensity level as a measure of DNA content, which is then correlated with the fluorescence intensity from different histone marks to measure the "dynamic" changes of histone marks during the cell cycle. This is the underlying basis of the method presented in this paper. This basis is correct only when the variation of the measured Hoechst intensity is due to the different cell cycle phases. However, as the authors used the objective with NA = 0.95, the depth of focus is only in the range of 1-2 microns. HeLa cells can be ~5-10um thick. Therefore, the effect of focal position should be carefully studied. For example, at a given focal plane, how many cells are in focus and how many cells are out of focus? How do authors distinguish "in-focus" from "out-of-focus" cells in an automatic fashion? Further, the authors need some validation step to confirm that their method of measuring DNA content (in terms of intensity distribution) with flow cytometry that is the gold standard in quantifying the DNA content.

We used a built-in auto-focus system to define the focal plane as a fixed distance. Fortunately, the HeLa and hTERT-RPE1 cells used in the study have only small variations in equatorial nuclear planes among cells under subconfluent conditions. When we compared several objective lenses, similar results were obtained using lenses with NA of 0.75–1.25. However, Hoechst intensities were broader with use of an oil-immersion lens (NA 1.4). These results have been included in the manuscript (p. 7–8 and 16; new Fig. 2 and 3).

We also confirmed the cell cycle profiles by FACS using samples stained with the same setup as that in Fig. 1 (Hoechst and three antibodies). These results have been presented in new Fig. S2A along with a description in the manuscript (p. 5).

(2) The authors presented Hoechst fluorescence intensity in a linear scale, but the intensity from histone marks in a logarithmic scale. It is not clear why they want to present the data this way, as these two are not power-law dependence.

In the original manuscript, we used log scales for histones because drastic changes were observed with some modifications (e.g., H4K20me1 and H4K5ac) during the cell cycle and perturbation assays (i.e., KDM expression and HDAC inhibitor treatments). However, we agree that most histone methylation fluctuates within 2-fold during the cell cycle, consistent with DNA. Thus, we agree on changing the plots to linear scale as suggested (Fig. 1, 2, 3, 4, S1A, S2D,E, and S4).

© 2020. Published by The Company of Biologists under the terms of the Creative Commons Attribution License (https://creativecommons.org/licenses/by/4.0/).

(3) I wonder whether the results remain robust if another DNA binding dye is used.

Hoechst 33342 has a preference to bind adenine-thymine (A-T) regions of DNA. Validation for at least Figure 1 or 2 using another dye will further validate their findings.

Per this suggestion, we have now compared the profiles of Hoechst and propidium iodide (PI), a DNA intercalator with little or no sequence preference, combined with RNase treatment (new Fig. S2D,E). The profiles of H4K20me1 (Alexa Fluor 488) and H4K5ac (Cy5) were found to be similar in cells stained with either Hoechst or PI. These results have been presented in new Fig. S2 with a description in the manuscript (p. 5).

(4) The authors did show the repeatability from 3 replicates. What is the impact of the various antibody efficiency?

We compared the profiles of single and double antibody staining to determine if double staining causes interference. We determined that the single and double labeling profiles were highly similar (new Fig. 2A). We also compared different antibody concentrations and obtained similar results (new Fig. S4). These results suggest that the system is considerably robust, which has been described in the revised manuscript (p. 6–7).

(5) There are a few technical components missing in this paper. Given this is presented as a method paper, the throughput of this method needs to be discussed. Further, cell nuclei segmentation is an important step in the image analysis. More detailed should be discussed. Is it fully automated or based on manual selection?

The cell nuclei are selected using semi-manual thresholding using NIS Elements. Once set in a single image, the same setting is applied to other images taken in the same sample. This procedure has been described in more detail in the Methods section (p. 20). The time taken for image analysis has also been described (p. 20) and the throughput has been mentioned on in the Discussion section (p. 17). We have also provided a link to a github website where the Mathematica code for plotting of the data has been uploaded (p. 21–22).

Reviewer 3 Comments for the Author:

(1) Although the presented method is based on a high throughput imaging and thus employs small objective lense (40x), to demonstrate a quality of immunostainings, images of at least several histone modifications have to be shown at a higher modification – with at least 63x and preferably as optical mid-nuclear sections. This should allow to see differential distribution of different modifications and thus to prove specificity and quality of immunostaining

We have compared the samples using different objective lenses [20× (NA 0.75) to 100× (NA 1.4)] to address Reviewer 1’s comment (3). We have also included magnified images to indicate the intranuclear distributions of individual modifications. Images captured using a 40× (NA 0.95) objective lens were considerably informative for distinct distributions, although those with higher NA had better clarity. These data have been described in the revised manuscript (p. 7–8 and new Figs. 2 and 3).

(2) Figure 1F: for clarity, direction from G2 to G1 should be added to the heat map on the right

As suggested, we have added indications for G1, S, and G2 in Fig. 1F. We have reordered the Hoechst intensity from the lowest to highest, starting with G1 from the top.

(3) Classification of modifications into three groups seems to me somewhat subjective: e.g., why H4K16ac belong to the second and not to the first group?

To clarify our classifications, we have rephrased the sentence describing the classification as “Based on the correlations with H4K5ac, we classified histone modifications into three groups for the sake of argument” (p. 9). The dynamics of H4K16ac were different from those of other acetylations. Most acetylations increased during the S phase and showed much higher correlations with H4K5ac

© 2020. Published by The Company of Biologists under the terms of the Creative Commons Attribution License (https://creativecommons.org/licenses/by/4.0/).

than with H4K16ac.

(4) Figure 2C: the plot looks very busy and difficult to read; it would be more clear if the three groups of modifications are color-coded and the font of the lettering isreduced

As suggested, each group has been color-coded and the lettering size has been reduced in new Fig. 4C (original Fig. 2C).

(5) Fig.3C: markings “n=400” and “top50” have to be added to columns, as they stand in 3B.

As suggested, these markings have been added to new Fig. 5C (original Fig. 3C).

(6) M&M: throughout the paper, the authors indicate Hochst as the nuclear stain, however, in the description of the used microscope they indicate a filter for DAPI. What was used - DAPI or Hoechst?

Thank you for pointing out this error. We only used Hoechst and this has been corrected in the manuscript (p. 20).

Second decision letter

MS ID#: JOCES/2019/243444

MS TITLE: Histone modification dynamics as revealed by a multicolor immunofluorescence-based single-cell analysis

AUTHORS: Yoko Hayashi-Takanaka, Yuto Kina, Fumiaki Nakamura, Leontine E Becking, Yoichi Nakao, Takahiro Nagase, Naohito Nozaki, and Hiroshi Kimura
ARTICLE TYPE: Tools and Resources

I am happy to tell you that your manuscript has been accepted for publication in Journal of Cell Science, pending standard ethics checks.

Reviewer 1

Advance summary and potential significance to field

They have developed a relatively high throughput single cell based assay for quantifying histone modifications and their relationship to the cell cycle.
They demonstrated the utility of this assay by screening for compounds that inhibit histone deacetylases and extensively validated the accuracy of the assay by reproducing cell cycle dynamics and specificities of histone demethylases. I think that this is a well documented, easy to implement assay such that it can be an important tool in the epigenetics toolbox.

Comments for the author

Overall, the authors went beyond my expectations in addressing my concerns.
The sensitivity of the antibodies to adjacent modifications, particularly mitotic phosphorylation, now clearly stated in the manuscript, provides an appropriate rationale for excluding mitotic cells.
I thought that the analysis of competition between antibodies and the extended analysis of the impact of numerical aperture on the performance of the assay provide an important resource for those trying to implement the approach using different instruments and without significant experience.

© 2020. Published by The Company of Biologists under the terms of the Creative Commons Attribution License (https://creativecommons.org/licenses/by/4.0/).

Reviewer 2

Advance summary and potential significance to field

This manuscript presented a method using multi-color immunofluorescence and DNA staining to image a large set of histone modifications during cell cycle and its application to screen a set of compounds. This approach provides a tool for in situ analysis of histone modifications that could be useful to the cell biology community.

Comments for the author

I think the authors addressed my previous questions. I now recommend the paper for publication.

© 2020. Published by The Company of Biologists under the terms of the Creative Commons Attribution License (https://creativecommons.org/licenses/by/4.0/).
